# Community Forests and Public Health: A Research Agenda

**DOI:** 10.3390/ijerph22101601

**Published:** 2025-10-21

**Authors:** Pooja S. Tandon, Shelby Semmes, Kim Garrett, Liv Ellerton, Susan Charnley, Howard Frumkin

**Affiliations:** 1 Trust for Public Land, San Francisco, CA 94108, USA; shelby.semmes@tpl.org (S.S.); frumkin@uw.edu (H.F.); 2 Department of Pediatrics, School of Medicine, University of Washington, Seattle, WA 98195, USA; 3 Seattle Children’s Research Institute, Seattle, WA 98101, USA; kim.garrett@seattlechildrens.org (K.G.); liv.ellerton@seattlechildrens.org (L.E.); 4 School of Public Health, University of Washington, Seattle, WA 98105, USA; 5 College of Forestry, Oregon State University, Corvallis, OR 97331, USA; susan.charnley@oregonstate.edu; 6 U.S. Forest Service, Corvallis, OR 97331, USA; 7 School of Public Health, Texas A&M University, College Station, TX 77840, USA

**Keywords:** greenspace access, rural health, community health, forest benefits

## Abstract

The natural environment is integral to supporting healthy and resilient communities. Community forests (CFs) are forested parcels, typically in rural areas, where community members have access, share governance, and receive various benefits. While considerable research demonstrates that urban parks and forests are important for human health, similar assessments are less available for CFs specifically. Although CFs exist in multiple countries, their policy, ecological, ownership, and governance contexts differ significantly. This review focuses on CFs in the United States. The goals of this project were to systematically review current evidence on the relationship between CFs and human health, identify knowledge gaps in the existing research, and propose a scientific research agenda that identifies critical questions related to CFs and public health in the U.S., with application in other contexts. We conducted a systematic review of the literature, screening 351 studies and assessing twenty-four full-text articles, only one of which met inclusion criteria. This mixed-methods study characterized 70 CFs in the Eastern U.S. and featured four case studies. The majority of CFs (93%) and all case studies identified recreational use as their most common purpose. The evidence base on the health implications of CFs is very thin. Targeted research on CFs and their impact on health could provide evidence to inform CF processes and help optimize their health outcomes. We propose a research agenda on CFs in the U.S. based on several pathways of public health promotion: nature contact, climate mitigation/adaptation, economic opportunities, community cohesion, and equity.

## 1. Introduction

A thriving natural world is increasingly seen as a vital component of healthy and resilient communities [1]. A significant body of research supports the relationship between nature contact and human health across the lifecourse [2,3,4]. These benefits include improved physical and mental health through pathways such as increased physical activity, reduced stress, enhanced social connection, and lowered risk of chronic disease. Much of the research has focused on urban areas and demonstrates a strong association between urban nature, especially parks and tree canopy, and human well-being [5,6,7]. Assessment of similar effects is less available for rural areas, and studies that have examined both environments have found that there may be differences in benefits experienced from nature contact by those living in rural vs. urban communities [8,9,10]. More specifically, community forests (CFs) are nature-rich environments, typically located in rural communities, that could provide many direct and indirect health benefits. However, their health benefits have not previously been a focus of discussion.

CFs are defined as forests that are owned by or on behalf of a local community, where members have long-term access and derive benefits from the forest. They are managed and governed with meaningful community involvement and protect conservation values while allowing sustainable uses [11,12]. For example, in the U.S., the U.S. Forest Service (USFS) has a Community Forest Program that provides financial support for land acquisition to establish CFs that are owned by local or tribal governments or NGOs, provide public access, and are actively managed to deliver local community benefits under a CF management plan.

In this paper, our working definition of CFs aligns with this U.S. model [12]. CFs have been growing in number in the U.S. and internationally since the 1990s, particularly in the Global South. The literature from international settings focuses primarily on their conservation and economic benefits [13,14]. CF models vary widely across countries in terms of governance, ownership, legal frameworks, and cultural meanings. Notably, the definition of community also differs across countries and contexts, varying in terms of population size, spatial distribution, infrastructure, and social organization. This variability may make cross-context generalizations challenging; nevertheless, there are likely common public health benefits of CFs, which can be best understood within their particular geographic contexts.

There are at least 98 CFs in the U.S., concentrated in New England and the Pacific Northwest [15]. CFs in the U.S. are distinct from urban forests, which are located within cities and managed by municipal agencies primarily to maintain and improve trees and forests that provide shade, recreation, green space, and other ecosystem services to urban residents. They also differ from commercial forests, which are usually owned by private corporations and managed for timber production to generate profits for their owners (e.g., shareholders, investors) as their primary beneficiaries, often with limited public access and no input from local residents.

While urban and CFs differ in governance and context, the mechanisms by which green spaces support human health offer valuable insights for understanding the potential of CFs to improve public health. Studies have shown that urban forests improve air quality by reducing pollutants like PM_2.5_ and NO_2_, help mitigate urban heat, and encourage physical activity, which can lower rates of obesity and cardiovascular disease [16]. They also support mental health by reducing stress, anxiety, and depression, and enhancing cognitive function [17,18]. Urban forests are linked to a range of public health benefits, including lower rates of cardiovascular and non-accidental mortality, reduced crime, and improved birth outcomes [19,20].

Just as urban forests serve as a cost-effective public health intervention, CFs may offer similar benefits in rural settings [21]. CFs have been recognized for their potential to help communities thrive [22]. CFs can offer a wide range of benefits to nearby residents by serving as settings for nature exposure, educational and recreational activities, and providing economic opportunities from timber and non-timber forest products. There is also evidence of environmental benefits such as protecting biodiversity, storing carbon, improving forest health, and protecting water supplies [23,24]. It is likely that environmental protection, community cohesion, and economic vitality promote individual and public health in nearby communities, but these pathways have not been well studied. Therefore, the goal of this project was to systematically review and synthesize current evidence on the relationship between CFs and human health, identify knowledge gaps in existing research, and propose a scientific research agenda that identifies critical questions related to CFs and public health in the U.S., with application to other contexts.

## 2. Materials and Methods

The literature review was conducted following Preferred Reporting Items for Systematic Reviews and Meta-Analyses (PRISMA) guidelines [25] and was registered in Prospero (CRD42024501290).

### 2.1. Data Sources

We conducted a comprehensive search across multiple databases with the help of a University of Washington librarian. The initial search was performed using PubMed and Environment Complete in RIS format. Subsequent searches were conducted through PsychINFO, Greenfile, Web of Science Core Collection, ProQuest, and the Cumulative Index to Nursing and Allied Health Literature.

All searches included a concept term related to “community forest” and an outcome concept term related to “health”. Searches were conducted with broad terms to encompass the multiple ways health and well-being can be discussed, including (1) physical health (including health behaviors, e.g., nutrition, physical activity); (2) mental health (e.g., stress, anxiety, depression); (3) medical conditions (e.g., asthma, cancer, blood pressure); and (4) environmental or economic benefits with mention of health (e.g., clean water, educational opportunities, employment). Search strings were altered to fit each database (File S1). We excluded articles that (1) did not include human subjects; (2) were literature reviews; (3) did not fit our definition of CF; and (4) had outcomes not distinctly mentioning health or health related behaviors or outcomes. The definition of “community forest” included forests where the community members have access to the land and its resources, participate in decisions concerning the forests, and work towards protecting and restoring the forests while prioritizing benefits to local communities. Quantitative, qualitative and mixed-methods studies could be included. No date limits were applied, and all databases were searched from their inception through 21 April 2025.

### 2.2. Study Selection and Data Synthesis

Initial screening and selection of papers were conducted through Covidence, a web-based platform for managing literature reviews. The authors collaborated on the criteria for study inclusion and exclusion. Studies were first screened by two authors by their title and abstracts. Studies that made it through then had their full text screened by two authors for inclusion. Any conflict was remedied with discussion including a third author. We did not exclude international studies until the full-text review stage so that we could include relevant information in this review, even though the focus of the project is on CFs in the U.S.

We planned to extract key characteristics from included studies, including study design, geographic setting, population, CF governance and use, health-related exposures and outcomes, and main findings. Our analytic approach used a narrative synthesis, with the goal of identifying themes across studies and mapping findings onto hypothesized pathways between CFs and health (e.g., nature contact, economic opportunity, climate resilience, and social cohesion). However, as only one study ultimately met the inclusion criteria, this approach could not be fully implemented.

## 3. Results

**Figure 1:**
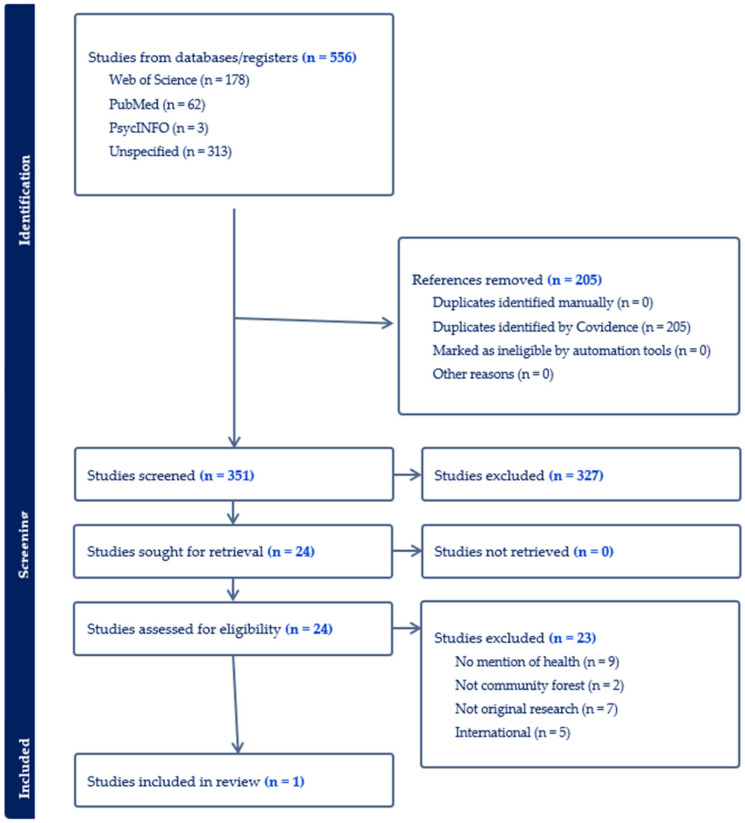
PRISMA Literature Flow Diagram.

Only one U.S.-based study met our inclusion criteria [26]. See Figure 1 for studies excluded at each stage. Five additional studies met other inclusion criteria but were based in countries outside of the U.S.; their full texts were reviewed, and a summary of findings is presented below. The single included study characterizes CFs in the Eastern U.S. in terms of forest ownership, governance, uses, and benefits [26]. The investigators used mixed methods to characterize 70 CFs and featured 4 case studies.

The CFs varied widely in size with a median area of 142 hectares. Most of the CFs were established with funding from land acquisition programs and managed by local nonprofit or municipal partnerships. The majority (93%) of CFs in this paper identify providing cultural ecosystem services, including recreational opportunities such as hiking, biking, snowmobiling, or horseback riding as a goal. Eighty-seven percent of the CF websites mention that they support ecosystem services for their community, such as clean water and air, carbon sequestration, and biodiversity. Other commonly noted purposes include education, and generation of timber and non-timber forest products. All four case studies had recreational use as their most common goal, and they all describe their CF playing an ecological role that benefits the community. While specific health outcomes were not explicitly addressed in this paper, health was addressed more broadly. The examples provided for recreational use suggest that CFs were providing opportunities for physical activity, which is an important health-promoting behavior. References to the role of CFs in supporting clean water and air also suggest environmental benefits that have implications for human health. The study’s findings are limited by the relatively small number of case studies (4 cases based on 18 interviews), reliance on online searches for the quantitative data, and the lack of an explicit focus on health outcomes.

Although excluded from our review due to its U.S. focus, five international studies offer some insights into how CFs may influence health and well-being owing to explicit mention of health. Studies from the UK highlight how access to and perceptions of green space in CFs relate to public health, especially if designed and programmed with cross-sector collaboration [27,28]. In the Pinder et al. study, residents perceived a range of health benefits including physical health and emotional well-being through their engagement with the forest. The study emphasizes that perceptions of safety, accessibility, and personal connection to place are key factors in how CFs support health. Rasolofoson et al. showed that conservation interventions in Madagascar, including CFs, can impact subjective well-being, especially when locally defined measures are used [29]. Loveridge et al. found that certified CFs in Tanzania supported both ecological goals and human well-being [30]. In Canada, Teitelbaum highlighted active recreational opportunities in CFs such as hiking and snow sports [31]. Collectively, these studies support the idea that CFs can promote health through various pathways, although none of them directly measured health outcomes.

## 4. Discussion

This attempt to systematically review peer-reviewed the scientific literature on the direct relationship between CFs and health in the U.S. yielded only one study. Even this study did not have explicit health aims, but was included because it identified physically active recreation, a health promoting behavior, as a primary use of CFs. It also referenced environmental health benefits conferred by forests through clean air and water. This study also did not include direct measurement of health outcomes, which limits its utility for drawing health-specific conclusions. This gap highlights the need for future studies that incorporate health data, either through primary data collection or linkage to health surveillance systems, and that explicitly test hypotheses about the health impacts of CFs.

Research on this topic remains sparse for several potential reasons. CFs are relatively few and geographically concentrated, and typically emphasize conservation and local economic development, rather than health. Attributing health benefits to CF exposure also poses methodological challenges that require longitudinal and multilevel designs. These demands, combined with limited research priority and funding, have likely contributed to the absence of a robust literature base. Therefore, there is considerable opportunity to study the potential of CFs to promote individual and community health, particularly for rural communities. Taking lessons from the broader literature on nature and health [32,33], especially the substantial evidence on the health promoting benefits of parks [5,7], future studies could better delineate the range of health outcomes that could be affected by CFs and under what circumstances the most health benefits emerge. Future studies could also draw on local and community knowledge to complement the limited peer-reviewed evidence and deepen understanding of how CFs affect health and well-being.

CFs represent a unique, underrecognized asset for promoting public health in rural areas of the U.S., where structural health disparities, including limited access to recreation, health care, and climate resilience infrastructure persist. The extant literature on nature and health suggests several pathways through which CFs may deliver health benefits. First, direct and indirect contact with nature promotes health, and CFs offer unique access, especially in some rural areas [34,35,36]. Second, the role of CFs in mitigating and adapting to climate change may contribute to health benefits [37]. Third, economic benefits of CFs may translate to health benefits [29]. Fourth, CFs may function to strengthen community social bonds, which in turn promotes health. Fifth, CFs often directly address equity issues relating to forest access, use, and governance, which may have positive health benefits for underserved or marginalized populations.

We describe each of these below and propose a research agenda on CFs and health for the U.S. based on these pathways. Within each of these domains we highlight public health implications and suggest specific research questions. Importantly, we do not suggest “research for the sake of research”; instead, we attempt to identify research questions that, if answered, can guide real-world decisions about CF acquisition, management, and programming, to maximize the health benefits they deliver.

Some research agendas propose priorities to help guide research decisions. Here we have not done so, for three reasons. First, the essence of CFs is community control—and communities will vary in how they prioritize health, climate, economic, community-building, and equity questions. We believe research priorities are best selected at a local level to reflect local knowledge needs. Second, CFs deliver multiple benefits, and well-designed research will likely study multiple outcomes. As communities and their research partners plan studies, the question will likely not be “Which outcome should we study?”, but rather “How many of these desired outcomes can we quantify?” Finally, at a time of scarce research funding, communities and their research partners will need to take into account the priorities of research funders.

While many of these domains could apply to forests in rural areas generally, their governance by and connection with local communities, and management to prioritize local community benefits, uniquely position CFs to support human health. Access to forests has become increasingly difficult in some areas because of factors such as distance, paucity of public forested lands, and forest conversion to developed uses, making CFs an important strategy for increasing equitable access to this potentially health-promoting resource.

### 4.1. Direct Health Benefits

The list of evidence-based health benefits of nature contact is long, and includes both physical and mental health and well-being, but depends on having access to nature [32]. Forest bathing (*Shinrin-yoku*) refers to a healing technique that restores physical and psychological health through exposure to a forest environment [38,39]. Drawing on this evidence, CFs could serve as environments for health promotion and disease prevention through mechanisms such as reducing stress, lowering blood pressure, and strengthening immune function. Access to and time spent in a CF could lead to increased physical activity [40] which in turn is associated with healthier weight status, better sleep, improved cardiovascular outcomes, and numerous other benefits for people of all ages [41]. It could also decrease stress and improve symptoms of depression or anxiety. At a time when physical inactivity, obesity, and mental health issues are all public health priorities, it would be useful to document the role of CFs in promoting healthy behaviors and a range of health outcomes. In addition, research could focus on what aspects of CFs—e.g., proximity to where people live, programs such as walking clubs that encourage spending time in CFs, amenities such as trails, benches, and emergency call boxes—most effectively facilitate health benefits. Different populations (e.g., older adults, children, people with chronic conditions) may experience unique benefits and face distinct barriers, so understanding these differences would help tailor interventions. Relatedly, research could clarify what ownership models and which partnerships—say, with local schools, religious congregations, hospitals, or civic organizations—most effectively facilitate health benefits from CFs.

Time in CFs, like any time in natural settings, can also carry some health risks, ranging from falls to chainsaw injuries, from insect bites to excessive sun exposure. Future studies could document the incidence, risk factors, and effective prevention strategies for these risks in CF settings (Table 1).

### 4.2. Climate Impact

CFs could promote human health through their capacity as nature-based strategies for mitigation of and adaptation to climate change [37,42]. Forests sequester and store carbon, improve air and water quality, lower local temperatures during extreme heat, and help with stormwater management [37,43]. While many of these benefits are best documented in urban settings, they may also operate in rural areas. However, the scientific literature regarding rural climate benefits is less robust, especially with regard to the human health co-benefits of CF-based climate change mitigation [44,45]. Because CFs are small and locally based, they may be especially nimble in responding to climate-related disturbances such as wildfires and insects [46] (Table 2).

### 4.3. Economic Benefits

The positive economic impact of CFs for rural communities has been previously documented and derives from production of forest-based products (i.e., timber, firewood), non-timber goods (food, maple syrup) and services, and bolstering local economies through employment opportunities [47]. Protecting scenic quality and recreational opportunities may help support a tourist economy that includes outfitters, equipment sales and rental, guide services, hotels, restaurants, and other economic activity. However, increases in property values creates gentrification, posing challenges for workers who rely on recreation-based employment but may be priced out of nearby housing. Economic deprivation and poverty are of course related to health outcomes [48]. Compared to urban areas, people living in rural communities face higher poverty rates, lower educational attainment, and lack of access to health services, and numerous health disparities across their lifespan [49,50]. Children in rural areas experience higher rates of all-cause mortality, suicide, firearm-related unintentional injury, and obesity [51]. Therefore, any boost to the local economy has the potential to uplift the socioeconomic status of residents and promote health (Table 3).

### 4.4. Community Cohesion

Central to the concept of CFs is the goal of empowering and connecting a community to manage its local forest and building a sense of place there. There is considerable support for the idea that a society’s social fabric is related to individual and population level health outcomes [52]. Loneliness and social isolation have been identified as serious public health risks [53]. By design, CFs offer community members a voice in how local lands are stewarded over time—a platform for community engagement and collective decision-making [54]. They can also protect and elevate cultural and spiritual connections to the land and offer opportunities for education and recreation that could bring community members together. Urban parks serve as a similar nature-rich community resource where community members can gather, connect, and build trust– all of which is good for their health [55]. CFs have similar potential to support the health of the local community through building social capital but little or no research has documented this process (Table 4).

### 4.5. Equity Considerations

In all of the domains mentioned above, an important consideration is to explore whether and how CFs promote equitable access to and use of CFs by community members of different ages, genders, race/ethnicities, income/educational status, health status, abilities, and other demographic characteristics that may be relevant. Just as access to greenspace and parks is marked by disparities in urban areas, often related to structural injustices [56], there are likely important inequities in physical and psychological access to greenspaces in rural areas. Because of their emphasis on community engagement, democratic governance, and creating local community benefits, CFs may be places where equity considerations are directly addressed (Table 5).

## 5. Conclusions

A systematic literature search revealed only one published paper from the U.S. indirectly linking CFs to individual or community health; this occurred by providing recreation opportunities. However, CFs, with their proximity to and connection with local communities, have considerable potential to promote physical and mental health in rural communities through several pathways. The pathways identified here are nature contact, climate impacts, economic opportunities, community cohesion, and increasing equity. There is a need for more research into CFs and their direct impact on health outcomes that can extend the broader research base for the health benefits of nature contact. Given the growing number of CFs in the U.S., it is crucial for future research to examine their role in promoting public health, particularly in underserved rural communities. While the only study that met our inclusion criteria focused on CFs in the Eastern U.S., the conceptual pathways and research agenda proposed in this review are not geographically limited. Rather, they are intended to guide future inquiry into how CFs across diverse contexts may support public health across the U.S. The research agenda can also be adapted for international studies of CFs and public health. As CFs continue to emerge and evolve in different parts of the U.S. and globally, understanding their potential contributions to health equity and rural well-being remains a critical area for interdisciplinary research to inform future investments and policies.

## Figures and Tables

**Table 1:** Research Opportunities Regarding Direct Health Benefits of CFs.

Do CFs promote physical activity (PA)? What kinds of PA are most prevalent in CFs, how much PA is attained, and how does that differ by sociodemographic characteristics such as age, gender, and other factors? What features of CFs, programs, public education efforts, and partnership arrangements promote PA? Does recreational PA in CFs replace sedentary behaviors or replace PA in other settings such as gyms, sport courts, or other rural outdoor spaces?
2. Do CFs promote mental health? What kinds of mental health benefits do people experience from spending time in a CF? Does spending time in a CF improve mood, decrease stress, decrease symptoms of depression or anxiety? What features of CFs, programs, public education efforts, and partnership arrangements promote mental health benefits? How do mental health impacts differ by sociodemographic characteristics? By diagnostic categories? Can visits to CFs be a part of the treatment plan for those living with mental illnesses such as depression, anxiety, substance use disorders, PTSD, problematic media use, and other diagnoses?
3. How can international evidence on the benefits of forest bathing be adapted and studied in CF settings?
4. How can those who live nearby be encouraged to spend more time in CFs? What barriers and facilitating factors exist for recreational use of CFs by the local community? How do these differ by sociodemographic characteristics? How could rural hospitals, clinics, or clinicians encourage time in CFs using nature prescriptions or other similar initiatives? How could schools and other youth-serving organizations support youth in spending time in CFs?
5. What is the impact on health outcomes of interventions designed to increase visitation to CFs?

**Table 2:** Research Opportunities Regarding CFs and Health due to Climate Impact.

What climate services (i.e., carbon sequestration, heat reduction, water storage and purification and stormwater management) are provided by CFs in qualitative and quantitative terms, and what are their human health co-benefits?
2. What are the local economic implications of these climate services?

**Table 3:** Research Opportunities Regarding CFs and Health due to Economic Benefits.

To what extent do CFs contribute to local economic development, and what are the patterns of this economic development (e.g., which groups benefit)?
2. What are the mechanisms through which an improving rural economy leads to improvements in individual and population health? What interventions optimize these positive health outcomes?

**Table 4:** Research Opportunities Regarding CFs and Health due to Community Cohesion.

What is the impact of CFs on outcomes related to social cohesion and sense of place for those living in the community?
2. What role does programming/establishing CFs/CF governance play in fostering social cohesion?
3. How does a sense of social cohesion relate to health outcomes for rural communities?

**Table 5:** Research Opportunities Regarding CFs and Health Equity Considerations.

How do CFs specifically address equity considerations through their governance and management institutions?
2. What disparities exist in governance of, access to, and use of CFs for recreational and other purposes based on various sociodemographic characteristics?
3. What programs, initiatives, and/or policies are successful in increasing equitable access to and use of CFs?

## Supplementary Materials

The following supporting information can be downloaded at https://www.mdpi.com/article/10.3390/ijerph22101601/s1, File S1: Search Terms.

## References

[1] Seven Vital Conditions for Health and Well-Being. https://www.communitycommons.org/collections/Seven-Vital-Conditions-for-Health-and-Well-Being.

[2] Jimenez M.P., DeVille N.V., Elliott E.G., Schiff J.E., Wilt G.E., Hart J.E., James P. (2021). Associations between Nature Exposure and Health: A Review of the Evidence. Int. J. Env. Res. Public Health.

[3] Besser L., Lovasi G. (2023). Human physical health outcomes influenced by contact with nature. Nature-Based Solutions for Cities.

[4] Konijnendijk C., Devkota D., Mansourian S., Wildburger C. (2023). Forests and Trees for Human Health: Pathways, Impacts, Challenges and Response Options.

[5] Gianfredi V., Buffoli M., Rebecchi A., Croci R., Oradini-Alacreu A., Stirparo G., Marino A., Odone A., Capolongo S., Signorelli C. (2021). Association between Urban Greenspace and Health: A Systematic Review of Literature. Int. J. Env. Res. Public Health.

[6] Pinto L.V., Inácio M., Ferreira C.S.S., Ferreira A.D., Pereira P. (2022). Ecosystem services and well-being dimensions related to urban green spaces–A systematic review. Sustain. Cities Soc..

[7] Reyes-Riveros R., Altamirano A., De La Barrera F., Rozas-Vásquez D., Vieli L., Meli P. (2021). Linking public urban green spaces and human well-being: A systematic review. Urban. For. Urban. Green..

[8] Bashan D., Colléony A., Shwartz A. (2021). Urban versus rural? The effects of residential status on species identification skills and connection to nature. People Nat..

[9] Ryan S.C., Sugg M.M., Runkle J.D., Matthews J.L. (2023). Spatial Analysis of Greenspace and Mental Health in North Carolina: Consideration of Rural and Urban Communities. Fam. Community Health.

[10] Lewis A., Devenish K., Dolan R., Garraty T., Okosun O., Scowen M., Welivita I., Willcock S., Misiune I., Depellegrin D., Egarter Vigl L. (2022). Ecosystem Service Flows Across the Rural-Urban Spectrum. Human-Nature Interactions: Exploring Nature’s Values Across Landscapes.

[11] Brendler T., Carey H. (1998). Community Forestry, Defined. J. For..

[12] Frey G.E., Hajjar R., Charnley S., McGinley K., Schelhas J., Tarr N.A., McCaskill L., Cubbage F.W. (2024). “Community Forests” in the United States–How Do we Know One When We See One?. Soc. Nat. Resour..

[13] Kaushal K.K., Melkani V.K., Kala J.C. (2005). Sustainable poverty alleviation through a forestry project in Tamilnadu State of India. Int. J. Sustain. Dev. World Ecol..

[14] Belsky J.M. (2015). Community forestry engagement with market forces: A comparative perspective from Bhutan and Montana. For. Policy Econ..

[15] Hajjar R., McGinley K., Charnley S., Frey G.E., Hovis M., Cubbage F.W., Schelhas J., Kornhauser K. (2024). Characterizing Community Forests in the United States. J. For..

[16] Bang K.S., Lee I.S., Kim S.J., Song M.K., Park S.E. (2016). The Effects of Urban Forest-walking Program on Health Promotion Behavior, Physical Health, Depression, and Quality of Life: A Randomized Controlled Trial of Office-workers. J. Korean Acad. Nurs..

[17] Wolf K.L., Lam S.T., McKeen J.K., Richardson G.R.A., van den Bosch M., Bardekjian A.C. (2020). Urban Trees and Human Health: A Scoping Review. Int. J. Environ. Res. Public Health.

[18] Giacinto J.J., Fricker G.A., Ritter M., Yost J., Doremus J. (2021). Urban forest biodiversity and cardiovascular disease: Potential health benefits from California’s street trees. PLoS ONE.

[19] Kirkland J., Donovan G. (2011). Growing Quality of Life: Urban Trees, Birth Weight, and Crime.

[20] Donovan G.H., Butry D.T., Michael Y.L., Prestemon J.P., Liebhold A.M., Gatziolis D., Mao M.Y. (2013). The relationship between trees and human health: Evidence from the spread of the emerald ash borer. Am. J. Prev. Med..

[21] Donovan G.H. (2017). Including public-health benefits of trees in urban-forestry decision making. Urban For. Urban Green..

[22] Charnley S., Poe M.R. (2007). Community Forestry in Theory and Practice: Where Are We Now?. Annu. Rev. Anthropol..

[23] Fischer H.W., Chhatre A., Duddu A., Pradhan N., Agrawal A. (2023). Community forest governance and synergies among carbon, biodiversity and livelihoods. Nat. Clim. Change.

[24] Liu N., Dobbs G.R., Caldwell P.V., Miniat C.F., Sun G., Duan K., Nelson S.A.C., Bolstad P.V., Carlson C.P. (2022). Quantifying the Role of National Forest System and other Forested Lands in Providing Surface Drinking Water Supply for the Conterminous United States.

[25] PRISMA Statement. https://www.prisma-statement.org.

[26] Hovis M., Frey G., McGinley K., Cubbage F., Han X., Lupek M. (2022). Ownership, Governance, Uses, and Ecosystem Services of Community Forests in the Eastern United States. Forests.

[27] Pinder R., Kessel A., Green J., Grundy C. (2009). Exploring perceptions of health and the environment: A qualitative study of Thames Chase Community Forest. Health Place.

[28] Kessel A., Green J., Pinder R., Wilkinson P., Grundy C., Lachowycz K. (2009). Multidisciplinary research in public health: A case study of research on access to green space. Public Health.

[29] Rasolofoson R.A. (2024). Access to Human Health Benefits of Forests in Rural Low and Middle-Income Countries: A Literature Review and Conceptual Framework. Challenges.

[30] Loveridge R., Marshall A.R., Pfeifer M., Rushton S., Nnyiti P.P., Fredy L., Sallu S.M. (2022). Pathways to win-wins or trade-offs? How certified community forests impact forest restoration and human wellbeing. Philos. Trans. R. Soc. B Biol. Sci..

[31] Teitelbaum S. (2014). Criteria and indicators for the assessment of community forestry outcomes: A comparative analysis from Canada. J. Environ. Manag..

[32] Frumkin H., Bratman G.N., Breslow S.J., Cochran B., Kahn P.H., Lawler J.J., Levin P.S., Tandon P.S., Varanasi U., Wolf K.L. (2017). Nature Contact and Human Health: A Research Agenda. Environ. Health Perspect..

[33] Fyfe-Johnson A.L., Hazlehurst M.F., Perrins S.P., Bratman G.N., Thomas R., Garrett K.A., Hafferty K.R., Cullaz T.M., Marcuse E.K., Tandon P.S. (2021). Nature and Children’s Health: A Systematic Review. Pediatrics.

[34] Andersen L., Corazon S.S., Stigsdotter U.K. (2021). Nature exposure and its effects on immune system functioning: A systematic review. Int. J. Environ. Res. Public Health.

[35] Kuo M. (2015). How might contact with nature promote human health? Promising mechanisms and a possible central pathway. Front. Psychol..

[36] Repke M.A., Berry M.S., Iii L.G.C., Metcalf A., Hensen R.M., Phelan C. (2018). How does nature exposure make people healthier?: Evidence for the role of impulsivity and expanded space perception. PLoS ONE.

[37] van den Bosch M., Bartolomeu M.L., Williams S., Basnou C., Hamilton I., Nieuwenhuijsen M., Pino J., Tonne C. (2024). A scoping review of human health co-benefits of forest-based climate change mitigation in Europe. Environ. Int..

[38] Li Q. (2022). Effects of forest environment (Shinrin-yoku/Forest bathing) on health promotion and disease prevention—The Establishment of “Forest Medicine”—. Environ. Health Prev. Med..

[39] Hansen M.M., Jones R., Tocchini K. (2017). Shinrin-Yoku (Forest Bathing) and Nature Therapy: A State-of-the-Art Review. Int. J. Environ. Res. Public Health.

[40] Maddock J.E., Frumkin H. (2025). Physical Activity in Natural Settings: An Opportunity for Lifestyle Medicine. Am. J. Lifestyle Med..

[41] Office of the Assistant Secretary for Health (2018). Physical Activity Guidelines for Americans.

[42] Villarreal-Rosas J., Rhodes J.R., Sonter L.J., Possingham H.P., Vogl A.L. (2023). Optimal allocation of nature-based solutions to achieve climate mitigation and adaptation goals. People Nat..

[43] Urban Forests and Climate Change|Climate Change Resource Center. https://www.climatehubs.usda.gov/sites/default/files/Urban-Forests_CCRC.pdf.

[44] Novick K.A., Keenan T.F., Anderegg W.R.L., Normile C.P., Runkle B.R.K., Oldfield E.E., Shrestha G., Baldocchi D.D., Evans M.E.K., Randerson J.T. (2024). We need a solid scientific basis for nature-based climate solutions in the United States. Proc. Natl. Acad. Sci. USA.

[45] Buma B., Gordon D.R., Kleisner K.M., Bartuska A., Bidlack A., DeFries R., Ellis P., Friedlingstein P., Metzger S., Morgan G. (2024). Expert review of the science underlying nature-based climate solutions. Nat. Clim. Change.

[46] Dickson-Hoyle S., Copes-Gerbitz K., Hagerman S.M., Daniels L.D. (2024). Community Forests advance local wildfire governance and proactive management in British Columbia, Canada. Can. J. For. Res..

[47] Lyman M.W., Grimm C., Evans J.R. (2014). Community forests as a wealth creation strategy for rural communities. Community Dev..

[48] Braveman P.A., Cubbin C., Egerter S., Williams D.R., Pamuk E. (2010). Socioeconomic Disparities in Health in the United States: What the Patterns Tell Us. Am. J. Public Health.

[49] Sosin A.N., Carpenter-Song E.A. (2024). Reimagining Rural Health Equity: Understanding Disparities And Orienting Policy, Practice, And Research in Rural America. Health Aff..

[50] Coughlin S.S., Clary C., Johnson J.A., Berman A., Heboyan V., Benevides T., Moore J., George V. (2019). Continuing Challenges in Rural Health in the United States. J. Env. Health Sci..

[51] Bettenhausen J.L., Winterer C.M., Colvin J.D. (2021). Health and Poverty of Rural Children: An Under-Researched and Under-Resourced Vulnerable Population. Acad. Pediatr..

[52] Holt-Lunstad J. (2022). Social Connection as a Public Health Issue: The Evidence and a Systemic Framework for Prioritizing the “Social” in Social Determinants of Health. Annu. Rev. Public Health.

[53] (2023). Our Epidemic of Loneliness and Isolation. https://www.hhs.gov/sites/default/files/surgeon-general-social-connection-advisory.pdf.

[54] McGinley K.A., Charnley S., Cubbage F.W., Hajjar R., Frey G.E., Schelhas J., Hovis M., Kornhauser K. (2022). Community forest ownership, rights, and governance regimes in the United States. Routledge Handbook of Community Forestry.

[55] Jennings V., Bamkole O. (2019). The relationship between social cohesion and urban green space: An avenue for health promotion. Int. J. Environ. Res. Public Health.

[56] Rigolon A. (2016). A complex landscape of inequity in access to urban parks: A literature review. Landsc. Urban. Plan..

