# Supplementary material for: Community Forests and Public Health: A Research Agenda
Source: Int J Environ Res Public Health. 2025 Oct 21;22(10):1601. doi: 10.3390/ijerph22101601 (PMC12562926; doi:10.3390/ijerph22101601)
Supplement: Supplementary file 1 [file ijerph-22-01601-s001.zip › ijerph-3761198-supplementary.pdf]

Search Strings

USA string – searched in title, abstract, keywords, and subject headings

"United States"
USA
U.S.A.
U.S.
Appalachia*
"Great Lakes"
mid-Atlantic-state*
mid-Atlantic-region*
middle-Atlantic-state*
middle-Atlantic-region*
Midwest*-US*
Midwest*-U.S*
Midwest*-state*
"Great Plains"
heartland
"New England"
Northeast*-US*
Northeast*-U.S*
Northeast*-state*
"Pacific Northwest"
Northwest*-US*
Northwest*-U.S*
Northwest*-state*
Pacific-state*
Southeast*-state*
Southeast*-region
Southeast*-US*
Southeast*-U.S*
southern-state*
southern-US*
southern-U.S*
southwest*-state*
southwest*-US*
southwest*-U.S*
"deep South"
"Black Belt"
"Rust Belt"
"District of Columbia"
"Washington DC"
Washington-D.C.
Alabama
Alaska
Arizona
Arkansas
"Little Rock"
California
"Los Angeles"
"San Diego"
"San Francisco"
Colorado
Connecticut
Delaware
Florida
Gainesville
Jacksonville
Tampa
Tallahassee
Georgia
Atlanta
Hawai*
Honolulu
Idaho
Illinois
Chicago
Indiana
Indianapolis
"West Lafayette"
Iowa
Kansas
Wichita
Kentucky
Louisiana
"New Orleans"
"Baton Rouge"
Shreveport
Maine
Orono
Maryland
Massachusetts
Boston
Harvard
Michigan
Detroit
"Ann Arbor"
"East Lansing"
Minnesota
Minneapolis
Rochester
Mississippi
Missouri
Montana
Missoula
Nebraska
Nevada
"Las Vegas"
"New Hampshire"
"New Jersey"
"New Mexico"
"New York"
"North Carolina"
"North Dakota"
Ohio
Cincinnati
Oklahoma
Oregon
Pennsylvania
Philadelphia
"Rhode Island"
"South Carolina"
"South Dakota"
Tennessee
Nashville
Memphis
Texas
Houston
Utah
Vermont
Virginia
Seattle
"West Virginia"
Wisconsin
Wyoming
Washington

PubMed

	**Concept: Community Forests**	**Concept: Health, ecosystem services, economic impacts**
Subject Headings (MeSH)	("Forests"[Mesh] AND ("Community Resources"[Mesh] OR "Community Networks"[Mesh] OR "Community Participation"[Mesh]))	Health<br>Economics
Free text terms (searched in [text words]() [tw] or title/abstract/author keywords with proximity searching [tiab:~0])	"community forest*"[tw]<br>"communal forest*"[tw]<br>"community-managed forest*"[tw]<br>"social forest*"[tw]<br>"local forest*"[tw]<br>"commons forest"[tiab:~0]<br>"commons forests"[tiab:~0]<br>"commons forestland"[tiab:~0]<br>"commons forestlands"[tiab:~0]<br>"commons forestry"[tiab:~0]<br>"town forest"[tiab:~0]<br>"town forests"[tiab:~0]<br>"town forestland"[tiab:~0]<br>"town forestlands"[tiab:~0]<br>"town forestry"[tiab:~0]<br>"city forest*"[tw]<br>"metropolitan forest"[tiab:~0]<br>"metropolitan forests"[tiab:~0]<br>"metropolitan forestland"[tiab:~0]<br>"metropolitan forestlands"[tiab:~0]<br>"metropolitan forestry"[tiab:~0]<br>"suburban forest*"[tw]<br>"nonurban forest"[tiab:~0]<br>"nonurban forests"[tiab:~0]<br>"nonurban forestland"[tiab:~0]<br>"nonurban forestlands"[tiab:~0]<br>"nonurban forestry"[tiab:~0]<br>"nonmetropolitan forest"[tiab:~0]<br>"nonmetropolitan forests"[tiab:~0]<br>"nonmetropolitan forestland"[tiab:~0]<br>"nonmetropolitan forestlands"[tiab:~0]<br>"nonmetropolitan forestry"[tiab:~0]<br>"community woodland"[tiab:~0]<br>"community woodland"[tiab:~0]<br>"communal woodland"[tiab:~0]<br>"community-managed woodland"[tiab:~0]<br>"social woodland"[tiab:~0]<br>"communal woodlands"[tiab:~0]<br>"community-managed woodlands"[tiab:~0]<br>"social woodlands"[tiab:~0]<br>"local woodland*"[tw]<br>"commons woodland"[tiab:~0]<br>"town woodland"[tiab:~0]<br>"commons woodlands"[tiab:~0]<br>"town woodlands"[tiab:~0]	health<br>well-being<br>wellbeing<br>prosperity<br>"economic opportunit*"<br>"economic contribution*"<br>"economic impact*"<br>"economic benefit*"<br>livelihood*<br>"ecosystem service*"

	"urban woodland*"[tw]<br>"city woodland"[tiab:~0]<br>"metropolitan woodland"[tiab:~0]<br>"city woodlands"[tiab:~0]<br>"metropolitan woodlands"[tiab:~0]<br>"suburban woodland*"[tw]<br>"nonurban woodland"[tiab:~0]<br>"nonmetropolitan woodland"[tiab:~0]<br>"nonurban woodlands"[tiab:~0]<br>"nonmetropolitan woodlands"[tiab:~0]<br>"forest commons"[tw]	

(("Forests"[Mesh] AND ("Community Resources"[Mesh] OR "Community Networks"[Mesh] OR "Community Participation"[Mesh])) OR "community forest*"[tw] OR "communal forest*"[tw] OR "community-managed forest*"[tw] OR "social forest*"[tw] OR "local forest*"[tw] OR "commons forest"[tiab:~0] OR "commons forests"[tiab:~0] OR "commons forestland"[tiab:~0] OR "commons forestlands"[tiab:~0] OR "commons forestry"[tiab:~0] OR "town forest"[tiab:~0] OR "town forests"[tiab:~0] OR "town forestland"[tiab:~0] OR "town forestlands"[tiab:~0] OR "town forestry"[tiab:~0] OR "city forest*"[tw] OR "metropolitan forest"[tiab:~0] OR "metropolitan forests"[tiab:~0] OR "metropolitan forestland"[tiab:~0] OR "metropolitan forestlands"[tiab:~0] OR "metropolitan forestry"[tiab:~0] OR "suburban forest*"[tw] OR "nonurban forest"[tiab:~0] OR "nonurban forests"[tiab:~0] OR "nonurban forestland"[tiab:~0] OR "nonurban forestlands"[tiab:~0] OR "nonurban forestry"[tiab:~0] OR "nonmetropolitan forest"[tiab:~0] OR "nonmetropolitan forests"[tiab:~0] OR "nonmetropolitan forestland"[tiab:~0] OR "nonmetropolitan forestlands"[tiab:~0] OR "nonmetropolitan forestry"[tiab:~0] OR "community woodland"[tiab:~0] OR "community woodland"[tiab:~0] OR "communal woodland"[tiab:~0] OR "community-managed woodland"[tiab:~0] OR "social woodland"[tiab:~0] OR "communal woodlands"[tiab:~0] OR "community-managed woodlands"[tiab:~0] OR "social woodlands"[tiab:~0] OR "local woodland*"[tw] OR "commons woodland"[tiab:~0] OR "town woodland"[tiab:~0] OR "commons woodlands"[tiab:~0] OR "town woodlands"[tiab:~0] OR "urban woodland*"[tw] OR "city woodland"[tiab:~0] OR "metropolitan woodland"[tiab:~0] OR "city woodlands"[tiab:~0] OR "metropolitan woodlands"[tiab:~0] OR "suburban woodland*"[tw] OR "nonurban woodland"[tiab:~0] OR "nonmetropolitan woodland"[tiab:~0] OR "nonurban woodlands"[tiab:~0] OR "nonmetropolitan woodlands"[tiab:~0] OR "forest commons"[tw]**)**

AND

(Health[Mesh] OR "Economics"[Mesh] OR health[tw] OR "well-being"[tw] OR wellbeing[tw] OR prosper*[tw] OR "economic opportunit*"[tw] OR "economic contribution*"[tw] OR "economic impact*"[tw] OR "economic benefit*"[tw] OR livelihood*[tw] OR "ecosystem service*"[tw]**)**

AND

("United States"[Mesh] OR "United States Government Agencies"[Mesh] OR "United States"[tw] OR USA[tw] OR U.S.A.[tw] OR U.S.[tw] OR Appalachia*[tw] OR "Great Lakes"[tw] OR mid-Atlantic-state*[tw] OR mid-Atlantic-region*[tw] OR middle-Atlantic-state*[tw] OR middle-Atlantic-region*[tw] OR Midwest*-US*[tw] OR Midwest*-U.S*[tw] OR Midwest*-state*[tw] OR "Great Plains"[tw] OR heartland[tw] OR "New England"[tw] OR Northeast*-US*[tw] OR Northeast*-U.S*[tw] OR Northeast*-state*[tw] OR "Pacific Northwest"[tw] OR Northwest*-US*[tw] OR Northwest*-U.S*[tw] OR Northwest*-state*[tw] OR Pacific-state*[tw] OR Southeast*-state*[tw] OR Southeast*-region[tw] OR Southeast*-US*[tw] OR Southeast*-U.S*[tw] OR southern-state*[tw] OR southern-US*[tw] OR southern-U.S*[tw] OR southwest*-state*[tw] OR southwest*-US*[tw] OR southwest*-U.S*[tw] OR "deep South"[tw] OR "Black Belt"[tw] OR "Rust Belt"[tw] OR "District of Columbia"[tw] OR "Washington DC"[tw] OR Washington-D.C.[tw] OR Alabama[tw] OR Alaska[tw] OR Arizona[tw] OR Arkansas[tw] OR "Little Rock"[tw] OR California[tw] OR "Los Angeles"[tw] OR "San Diego"[tw] OR "San Francisco"[tw] OR Colorado[tw] OR Connecticut[tw] OR Delaware[tw] OR Florida[tw] OR

Gainesville[tw] OR Jacksonville[tw] OR Tampa[tw] OR Tallahassee[tw] OR Georgia[tw] OR Atlanta[tw] OR Hawai*[tw] OR Honolulu[tw] OR Idaho[tw] OR Illinois[tw] OR Chicago[tw] OR Indiana[tw] OR Indianapolis[tw] OR "West Lafayette"[tw] OR Iowa[tw] OR Kansas[tw] OR Wichita[tw] OR Kentucky[tw] OR Louisiana[tw] OR "New Orleans"[tw] OR "Baton Rouge"[tw] OR Shreveport[tw] OR Maine[tw] OR Orono[tw] OR Maryland[tw] OR Massachusetts[tw] OR Boston[tw] OR Harvard[tw] OR Michigan[tw] OR Detroit[tw] OR "Ann Arbor"[tw] OR "East Lansing"[tw] OR Minnesota[tw] OR Minneapolis[tw] OR Rochester[tw] OR Mississippi[tw] OR Missouri[tw] OR Montana[tw] OR Missoula[tw] OR Nebraska[tw] OR Nevada[tw] OR "Las Vegas"[tw] OR "New Hampshire"[tw] OR "New Jersey"[tw] OR "New Mexico"[tw] OR "New York"[tw] OR "North Carolina"[tw] OR "North Dakota"[tw] OR Ohio[tw] OR Cincinnati[tw] OR Oklahoma[tw] OR Oregon[tw] OR Pennsylvania[tw] OR Philadelphia[tw] OR "Rhode Island"[tw] OR "South Carolina"[tw] OR "South Dakota"[tw] OR Tennessee[tw] OR Nashville[tw] OR Memphis[tw] OR Texas[tw] OR Houston[tw] OR Utah[tw] OR Vermont[tw] OR Virginia[tw] OR Seattle[tw] OR "West Virginia"[tw] OR Wisconsin[tw] OR Wyoming[tw] OR Washington[tw]**)**

Cumulative Index to Nursing and Allied Health Literature (CINAHL Complete); EBSCOhost

	Concept: Community Forests	Concept: Health, ecosystem services, economic impacts
Subject Headings (MH)	---	"Health+"<br>"Economics+"
Free text terms (searched in Title (TI), Abstract (AB), and Term in Subject Heading (MW); keyword not a searchable field)	"community forest*"<br>"communal forest*"<br>"community-managed forest*"<br>"social forest*"<br>"local forest*"<br>"commons forest*"<br>"town forest*"<br>"city forest*"<br>"metropolitan forest*"<br>"suburban forest*"<br>"nonurban forest*"<br>"nonmetropolitan forest*"<br>"community woodland*"<br>"communal woodland*"<br>"community-managed woodland*"<br>"social woodland*"<br>"local woodland*"<br>"commons woodland*"<br>"town woodland*"<br>"urban woodland*"<br>"city woodland*"<br>"metropolitan woodland*"<br>"suburban woodland*"<br>"nonurban woodland*"<br>"nonmetropolitan woodland*"<br>"forest commons"	health<br>well-being<br>wellbeing<br>prosperity<br>"economic opportunit*"<br>"economic contribution*"<br>"economic impact*"<br>"economic benefit*"<br>livelihood*<br>"ecosystem service*"

(TI("community forest*" OR "communal forest*" OR "community-managed forest*" OR "social forest*" OR "local forest*" OR "commons forest*" OR "town forest*" OR "city forest*" OR "metropolitan forest*" OR "suburban forest*" OR "nonurban forest*" OR "nonmetropolitan forest*" OR "community woodland*" OR "communal woodland*" OR "community-managed woodland*" OR "social woodland*" OR "local woodland*" OR "commons woodland*" OR "town woodland*" OR "urban woodland*" OR "city woodland*" OR "metropolitan woodland*" OR "suburban woodland*" OR "nonurban woodland*" OR "nonmetropolitan woodland*" OR "forest commons") OR AB("community forest*" OR "communal forest*" OR "community-managed forest*" OR "social forest*" OR "local forest*" OR "commons forest*" OR "town forest*" OR "city forest*" OR "metropolitan forest*" OR "suburban forest*" OR "nonurban forest*" OR "nonmetropolitan forest*" OR "community woodland*" OR "communal woodland*" OR "community-managed woodland*" OR "social woodland*" OR "local woodland*" OR "commons woodland*" OR "town woodland*" OR "urban woodland*" OR "city woodland*" OR "metropolitan woodland*" OR "suburban woodland*" OR "nonurban woodland*" OR "nonmetropolitan woodland*" OR "forest commons") OR MW("community forest*" OR "communal forest*" OR "community-managed forest*" OR "social forest*" OR "local forest*" OR "commons forest*" OR "town forest*" OR "city forest*" OR "metropolitan forest*" OR "suburban forest*" OR "nonurban forest*" OR "nonmetropolitan forest*" OR "community woodland*" OR "communal woodland*" OR "community-managed woodland*" OR "social woodland*" OR "local woodland*" OR "commons woodland*" OR "town woodland*" OR "urban woodland*" OR "city woodland*" OR "metropolitan woodland*" OR "suburban woodland*" OR "nonurban woodland*" OR "nonmetropolitan woodland*" OR "forest commons")**)**

AND

(MH("Health+" OR "Economics+") OR TI(health OR well-being OR wellbeing OR prosperity OR "economic opportunit*" OR "economic contribution*" OR "economic impact*" OR "economic benefit*" OR livelihood* OR "ecosystem service*") OR AB(health OR well-being OR wellbeing OR prosperity OR "economic opportunit*" OR "economic contribution*" OR "economic impact*" OR "economic benefit*" OR livelihood* OR "ecosystem service*") OR MW(health OR well-being OR wellbeing OR prosperity OR "economic opportunit*" OR "economic contribution*" OR "economic impact*" OR "economic benefit*" OR livelihood* OR "ecosystem service*")**)**

AND

((MH "United States+") OR SB "USA" OR

TI(“United States” OR USA OR U.S.A. OR U.S. OR Appalachia* OR “Great Lakes” OR mid-Atlantic-state* OR mid-Atlantic-region* OR middle-Atlantic-state* OR middle-Atlantic-region* OR "Midwest* US*" OR "Midwest* U.S*" OR Midwest*-state* OR “Great Plains” OR heartland OR "New England" OR "Northeast* US*" OR "Northeast* U.S*" OR Northeast*-state* OR “Pacific Northwest” OR "northwest* US*" OR "northwest* U.S*" OR Northwest*-state* OR Pacific-state* OR Southeast*-state* OR Southeast*-region OR "Southeast* US*" OR "Southeast* U.S*" OR southern-state* OR "southern US*" OR "southern U.S*" OR "Southwest* state*" OR "Southwest* US*" OR "southwest* U.S*" OR “deep South” OR “Black Belt” OR “Rust Belt” OR “District of Columbia” OR “Washington DC” OR Washington-D.C. OR Alabama OR Alaska OR Arizona OR Arkansas OR "Little Rock" OR California OR “Los Angeles” OR "San Diego" OR "San Francisco" OR Colorado OR Connecticut OR Delaware OR Florida OR Gainesville OR Jacksonville OR Tampa OR Tallahassee OR Georgia OR Atlanta OR Hawaii OR Hawai'i OR Hawai`i OR Honolulu OR Idaho OR Illinois OR Chicago OR Indiana OR Indianapolis OR "West Lafayette" OR Iowa OR Kansas OR Wichita OR Kentucky OR Louisiana OR “New Orleans” OR “Baton Rouge” OR Shreveport OR Maine OR Orono OR Maryland OR “johns Hopkins” OR Massachusetts OR Boston OR Harvard OR Michigan OR Detroit OR “Ann Arbor” OR “East Lansing” OR Minnesota OR Minneapolis OR Rochester OR Mississippi OR Missouri OR Montana OR Missoula OR Nebraska OR Nevada OR “Las Vegas” OR “New Hampshire” OR “New Jersey” OR “New Mexico” OR “New York” OR “North Carolina” OR “North Dakota” OR Ohio OR Cincinnati OR Oklahoma OR Oregon OR Pennsylvania OR Philadelphia OR “Rhode Island” OR “South Carolina” OR “South Dakota” OR Tennessee OR Nashville OR Memphis OR Texas OR Houston OR Utah OR Vermont OR Virginia OR Seattle OR “West Virginia” OR Wisconsin OR Wyoming OR Washington) OR AB(“United States” OR USA OR U.S.A. OR U.S. OR Appalachia* OR “Great Lakes” OR mid-Atlantic-state* OR mid-Atlantic-region* OR middle-Atlantic-state* OR middle-Atlantic-region* OR "Midwest* US*" OR "Midwest* U.S*" OR Midwest*-state* OR “Great Plains” OR heartland OR "New England" OR "Northeast* US*" OR "Northeast* U.S*" OR Northeast*-state* OR “Pacific Northwest” OR "northwest* US*" OR "northwest* U.S*" OR Northwest*-state* OR Pacific-state* OR Southeast*-state* OR Southeast*-region OR "Southeast* US*" OR "Southeast* U.S*" OR southern-state* OR "southern US*" OR "southern U.S*" OR "Southwest* state*" OR "Southwest* US*" OR "southwest* U.S*" OR “deep South” OR “Black Belt” OR “Rust Belt” OR “District of Columbia” OR “Washington DC” OR Washington-D.C. OR Alabama OR Alaska OR Arizona OR Arkansas OR "Little Rock" OR California OR “Los Angeles” OR "San Diego" OR "San Francisco" OR Colorado OR Connecticut OR Delaware OR Florida OR Gainesville OR Jacksonville OR Tampa OR Tallahassee OR Georgia OR Atlanta OR Hawaii OR Hawai'i OR Hawai`i OR Honolulu OR Idaho OR Illinois OR Chicago OR Indiana OR Indianapolis OR "West Lafayette" OR Iowa OR Kansas OR Wichita OR Kentucky OR Louisiana OR “New Orleans” OR “Baton Rouge” OR Shreveport OR Maine OR Orono OR Maryland OR “johns Hopkins” OR Massachusetts OR Boston OR Harvard OR Michigan OR Detroit OR “Ann Arbor” OR “East Lansing” OR Minnesota OR Minneapolis OR Rochester OR Mississippi OR Missouri OR Montana OR Missoula OR Nebraska OR Nevada OR “Las Vegas” OR “New Hampshire” OR “New Jersey” OR “New Mexico” OR “New York” OR “North Carolina” OR “North Dakota” OR Ohio OR Cincinnati OR Oklahoma OR Oregon OR Pennsylvania OR Philadelphia OR “Rhode Island” OR “South Carolina” OR “South Dakota” OR Tennessee OR Nashville OR Memphis OR Texas OR Houston OR Utah OR Vermont OR Virginia OR Seattle OR “West Virginia” OR Wisconsin OR Wyoming OR Washington) **)**

PsycInfo; EBSCOhost

	Concept: Community Forests	Concept: Health, ecosystem services, economic impacts
PsycInfo Subject Headings (DE)	---	"Adolescent Health"<br>"Cardiovascular Health"<br>"Disease Outbreaks"<br>"Emotional Health"<br>"Epidemiology"<br>"Global Health"<br>"Health Anxiety"<br>"Health Awareness"<br>"Health Behavior"<br>"Health Disparities"<br>"Health Outcomes"<br>"Health Status"<br>"Holistic Health"<br>"Occupational Health"<br>"Physical Health"<br>"Population Health"<br>"Public Health"<br>"Reproductive Health"<br>"Rural Health"<br>"Sexual Health"<br>"Well Being"<br>"Health"<br>"Spiritual Well Being"<br>"Subjective Well Being"<br>"Child Health"<br>"Mental Health"<br>"Social Health"<br>"Economics"
MeSH terms (MA)	(MA"Forests" AND (MA("Community Resources" OR "Community Networks" OR "Community Participation"))	"Health"<br>"Adolescent Health"<br>"Cardiorespiratory Fitness"<br>"Child Health"<br>"Family Health"<br>"Global Health"<br>"Holistic Health"<br>"Infant Health"<br>"Men's Health"<br>"Mental Health"<br>"Military Health"<br>"Minority Health"<br>"Occupational Health"<br>"One Health"<br>"Physical Fitness"<br>"Cardiorespiratory Fitness"<br>"Physical Functional Performance+"<br>"Population Health"<br>"Rural Health"<br>"Public Health"<br>"Reproductive Health"

		"Sexual Health"<br>"Veterans Health"<br>"Women's Health"<br>"Maternal Health"<br>"Economics"<br>"Economic Development"
Free text terms (searched in Title (TI), Abstract (AB), Keywords (KW) and Term in Subject Heading (SU)	"community forest*"<br>"communal forest*"<br>"community-managed forest*"<br>"social forest*"<br>"local forest*"<br>"commons forest*"<br>"town forest*"<br>"city forest*"<br>"metropolitan forest*"<br>"suburban forest*"<br>"nonurban forest*"<br>"nonmetropolitan forest*"<br>"community woodland*"<br>"communal woodland*"<br>"community-managed woodland*"<br>"social woodland*"<br>"local woodland*"<br>"commons woodland*"<br>"town woodland*"<br>"urban woodland*"<br>"city woodland*"<br>"metropolitan woodland*"<br>"suburban woodland*"<br>"nonurban woodland*"<br>"nonmetropolitan woodland*"<br>"forest commons"	health<br>well-being<br>wellbeing<br>prosperity<br>"economic opportunit*"<br>"economic contribution*"<br>"economic impact*"<br>"economic benefit*"<br>livelihood*<br>"ecosystem service*"

((MA"Forests" AND MA("Community Resources" OR "Community Networks" OR "Community Participation")) OR TI("community forest*" OR "communal forest*" OR "community-managed forest*" OR "social forest*" OR "local forest*" OR "commons forest*" OR "town forest*" OR "city forest*" OR "metropolitan forest*" OR "suburban forest*" OR "nonurban forest*" OR "nonmetropolitan forest*" OR "community woodland*" OR "communal woodland*" OR "community-managed woodland*" OR "social woodland*" OR "local woodland*" OR "commons woodland*" OR "town woodland*" OR "urban woodland*" OR "city woodland*" OR "metropolitan woodland*" OR "suburban woodland*" OR "nonurban woodland*" OR "nonmetropolitan woodland*" OR "forest commons") OR AB("community forest*" OR "communal forest*" OR "community-managed forest*" OR "social forest*" OR "local forest*" OR "commons forest*" OR "town forest*" OR "city forest*" OR "metropolitan forest*" OR "suburban forest*" OR "nonurban forest*" OR "nonmetropolitan forest*" OR "community woodland*" OR "communal woodland*" OR "community-managed woodland*" OR "social woodland*" OR "local woodland*" OR "commons woodland*" OR "town woodland*" OR "urban woodland*" OR "city woodland*" OR "metropolitan woodland*" OR "suburban woodland*" OR "nonurban woodland*" OR "nonmetropolitan woodland*" OR "forest commons") OR KW("community forest*" OR "communal forest*" OR "community-managed forest*" OR "social forest*" OR "local forest*" OR "commons forest*" OR "town forest*" OR "city forest*" OR "metropolitan forest*" OR "suburban forest*" OR "nonurban forest*" OR "nonmetropolitan forest*" OR "community woodland*" OR "communal woodland*" OR "community-managed woodland*" OR "social woodland*" OR "local woodland*" OR "commons woodland*" OR "town woodland*" OR "urban woodland*" OR "city woodland*" OR

"metropolitan woodland*" OR "suburban woodland*" OR "nonurban woodland*" OR "nonmetropolitan woodland*" OR "forest commons") OR SU("community forest*" OR "communal forest*" OR "community-managed forest*" OR "social forest*" OR "local forest*" OR "commons forest*" OR "town forest*" OR "city forest*" OR "metropolitan forest*" OR "suburban forest*" OR "nonurban forest*" OR "nonmetropolitan forest*" OR "community woodland*" OR "communal woodland*" OR "community-managed woodland*" OR "social woodland*" OR "local woodland*" OR "commons woodland*" OR "town woodland*" OR "urban woodland*" OR "city woodland*" OR "metropolitan woodland*" OR "suburban woodland*" OR "nonurban woodland*" OR "nonmetropolitan woodland*" OR "forest commons"))

AND

(DE("Adolescent Health" OR "Cardiovascular Health" OR "Disease Outbreaks" OR "Emotional Health" OR "Epidemiology" OR "Global Health" OR "Health Anxiety" OR "Health Awareness" OR "Health Behavior" OR "Health Disparities" OR "Health Outcomes" OR "Health Status" OR "Holistic Health" OR "Occupational Health" OR "Physical Health" OR "Population Health" OR "Public Health" OR "Reproductive Health" OR "Rural Health" OR "Sexual Health" OR "Well Being" OR "Health" OR "Spiritual Well Being" OR "Subjective Well Being" OR "Child Health" OR "Mental Health" OR "Social Health" OR "Economics") OR MA("Health" OR "Adolescent Health" OR "Cardiorespiratory Fitness" OR "Child Health" OR "Family Health" OR "Global Health" OR "Holistic Health" OR "Infant Health" OR "Men's Health" OR "Mental Health" OR "Military Health" OR "Minority Health" OR "Occupational Health" OR "One Health" OR "Physical Fitness" OR "Cardiorespiratory Fitness" OR "Physical Functional Performance+" OR "Population Health" OR "Rural Health" OR "Public Health" OR "Reproductive Health" OR "Sexual Health" OR "Veterans Health" OR "Women's Health" OR "Maternal Health" OR "Economics" OR "Economic Development") OR OR TI(health OR well-being OR wellbeing OR prosperity OR "economic opportunit*" OR "economic contribution*" OR "economic impact*" OR "economic benefit*" OR livelihood* OR "ecosystem service*") OR AB(health OR well-being OR wellbeing OR prosperity OR "economic opportunit*" OR "economic contribution*" OR "economic impact*" OR "economic benefit*" OR livelihood* OR "ecosystem service*") OR KW(health OR well-being OR wellbeing OR prosperity OR "economic opportunit*" OR "economic contribution*" OR "economic impact*" OR "economic benefit*" OR livelihood* OR "ecosystem service*") OR SU(health OR well-being OR wellbeing OR prosperity OR "economic opportunit*" OR "economic contribution*" OR "economic impact*" OR "economic benefit*" OR livelihood* OR "ecosystem service*")**)**

AND

(MA("United States" OR "Appalachian Region" OR "Great Lakes Region" OR "New England" OR "Mid-Atlantic Region" OR "Midwestern United States" OR "Northwestern United States" OR "Pacific States" OR "Southeastern United States" OR "Southwestern United States" OR Alabama OR Alaska OR Arizona OR Arkansas OR California OR Colorado OR Connecticut OR Delaware OR "District of Columbia" OR Florida OR Georgia OR Hawaii OR Idaho OR Illinois OR Indiana OR Iowa OR Kansas OR Kentucky OR Louisiana OR Maine OR Maryland OR Massachusetts OR Michigan OR Minnesota OR Mississippi OR Missouri OR Montana OR Nebraska OR Nevada OR "New Hampshire" OR "New Jersey" OR "New Mexico" OR "New York" OR "North Carolina" OR "North Dakota" OR Ohio OR Oklahoma OR Oregon OR Pennsylvania OR "Rhode Island" OR "South Carolina" OR "South Dakota" OR Tennessee OR Texas OR Utah OR Vermont OR Virginia OR Washington OR "West Virginia" OR Wisconsin OR Wyoming) OR TI("United States" OR USA OR U.S.A. OR U.S. OR Appalachia* OR "Great Lakes" OR mid-Atlantic-state* OR mid-Atlantic-region* OR middle-Atlantic-state* OR middle-Atlantic-region* OR "Midwest* US*" OR "Midwest* U.S*" OR Midwest*-state* OR "Great Plains" OR heartland OR "New England" OR "Northeast* US*" OR "Northeast* U.S*" OR Northeast*-state* OR "Pacific Northwest" OR "northwest* US*" OR "northwest* U.S*" OR Northwest*-state* OR Pacific-state* OR Southeast*-state* OR Southeast*-region OR "Southeast* US*" OR "Southeast* U.S*" OR southern-state* OR "southern US*" OR "southern U.S*" OR "Southwest* state*" OR "Southwest* US*" OR "southwest* U.S*" OR "deep South" OR "Black Belt" OR "Rust Belt" OR "District of Columbia" OR "Washington DC" OR Washington-D.C. OR Alabama OR Alaska OR Arizona OR Arkansas OR "Little Rock" OR California OR "Los Angeles" OR "San Diego" OR "San Francisco" OR Colorado OR Connecticut OR Delaware OR Florida OR Gainesville OR Jacksonville OR Tampa OR Tallahassee OR Georgia OR Atlanta OR Hawaii OR Hawai'i OR Hawai`i OR Honolulu OR Idaho OR Illinois OR Chicago OR Indiana OR Indianapolis OR "West Lafayette" OR Iowa OR Kansas OR Wichita OR Kentucky OR Louisiana OR "New Orleans" OR "Baton Rouge" OR Shreveport OR Maine OR Orono OR Maryland OR "johns Hopkins" OR Massachusetts OR Boston OR Harvard OR Michigan OR Detroit OR "Ann Arbor" OR

“East Lansing” OR Minnesota OR Minneapolis OR Rochester OR Mississippi OR Missouri OR Montana OR Missoula OR Nebraska OR Nevada OR “Las Vegas” OR “New Hampshire” OR “New Jersey” OR “New Mexico” OR “New York” OR “North Carolina” OR “North Dakota” OR Ohio OR Cincinnati OR Oklahoma OR Oregon OR Pennsylvania OR Philadelphia OR “Rhode Island” OR “South Carolina” OR “South Dakota” OR Tennessee OR Nashville OR Memphis OR Texas OR Houston OR Utah OR Vermont OR Virginia OR Seattle OR “West Virginia” OR Wisconsin OR Wyoming OR Washington) OR AB(“United States” OR USA OR U.S.A. OR U.S. OR Appalachia* OR “Great Lakes” OR mid-Atlantic-state* OR mid-Atlantic-region* OR middle-Atlantic-state* OR middle-Atlantic-region* OR "Midwest* US*" OR "Midwest* U.S*" OR Midwest*-state* OR “Great Plains” OR heartland OR "New England" OR "Northeast* US*" OR "Northeast* U.S*" OR Northeast*-state* OR “Pacific Northwest” OR "northwest* US*" OR "northwest* U.S*" OR Northwest*-state* OR Pacific-state* OR Southeast*-state* OR Southeast*-region OR "Southeast* US*" OR "Southeast* U.S*" OR southern-state* OR "southern US*" OR "southern U.S*" OR "Southwest* state*" OR "Southwest* US*" OR "southwest* U.S*" OR “deep South” OR “Black Belt” OR “Rust Belt” OR “District of Columbia” OR “Washington DC” OR Washington-D.C. OR Alabama OR Alaska OR Arizona OR Arkansas OR "Little Rock" OR California OR “Los Angeles” OR "San Diego" OR "San Francisco" OR Colorado OR Connecticut OR Delaware OR Florida OR Gainesville OR Jacksonville OR Tampa OR Tallahassee OR Georgia OR Atlanta OR Hawaii OR Hawai'i OR Hawai`i OR Honolulu OR Idaho OR Illinois OR Chicago OR Indiana OR Indianapolis OR "West Lafayette" OR Iowa OR Kansas OR Wichita OR Kentucky OR Louisiana OR “New Orleans” OR “Baton Rouge” OR Shreveport OR Maine OR Orono OR Maryland OR “johns Hopkins” OR Massachusetts OR Boston OR Harvard OR Michigan OR Detroit OR “Ann Arbor” OR “East Lansing” OR Minnesota OR Minneapolis OR Rochester OR Mississippi OR Missouri OR Montana OR Missoula OR Nebraska OR Nevada OR “Las Vegas” OR “New Hampshire” OR “New Jersey” OR “New Mexico” OR “New York” OR “North Carolina” OR “North Dakota” OR Ohio OR Cincinnati OR Oklahoma OR Oregon OR Pennsylvania OR Philadelphia OR “Rhode Island” OR “South Carolina” OR “South Dakota” OR Tennessee OR Nashville OR Memphis OR Texas OR Houston OR Utah OR Vermont OR Virginia OR Seattle OR “West Virginia” OR Wisconsin OR Wyoming OR Washington) OR KW(“United States” OR USA OR U.S.A. OR U.S. OR Appalachia* OR “Great Lakes” OR mid-Atlantic-state* OR mid-Atlantic-region* OR middle-Atlantic-state* OR middle-Atlantic-region* OR "Midwest* US*" OR "Midwest* U.S*" OR Midwest*-state* OR “Great Plains” OR heartland OR "New England" OR "Northeast* US*" OR "Northeast* U.S*" OR Northeast*-state* OR “Pacific Northwest” OR "northwest* US*" OR "northwest* U.S*" OR Northwest*-state* OR Pacific-state* OR Southeast*-state* OR Southeast*-region OR "Southeast* US*" OR "Southeast* U.S*" OR southern-state* OR "southern US*" OR "southern U.S*" OR "Southwest* state*" OR "Southwest* US*" OR "southwest* U.S*" OR “deep South” OR “Black Belt” OR “Rust Belt” OR “District of Columbia” OR “Washington DC” OR Washington-D.C. OR Alabama OR Alaska OR Arizona OR Arkansas OR "Little Rock" OR California OR “Los Angeles” OR "San Diego" OR "San Francisco" OR Colorado OR Connecticut OR Delaware OR Florida OR Gainesville OR Jacksonville OR Tampa OR Tallahassee OR Georgia OR Atlanta OR Hawaii OR Hawai'i OR Hawai`i OR Honolulu OR Idaho OR Illinois OR Chicago OR Indiana OR Indianapolis OR "West Lafayette" OR Iowa OR Kansas OR Wichita OR Kentucky OR Louisiana OR “New Orleans” OR “Baton Rouge” OR Shreveport OR Maine OR Orono OR Maryland OR “johns Hopkins” OR Massachusetts OR Boston OR Harvard OR Michigan OR Detroit OR “Ann Arbor” OR “East Lansing” OR Minnesota OR Minneapolis OR Rochester OR Mississippi OR Missouri OR Montana OR Missoula OR Nebraska OR Nevada OR “Las Vegas” OR “New Hampshire” OR “New Jersey” OR “New Mexico” OR “New York” OR “North Carolina” OR “North Dakota” OR Ohio OR Cincinnati OR Oklahoma OR Oregon OR Pennsylvania OR Philadelphia OR “Rhode Island” OR “South Carolina” OR “South Dakota” OR Tennessee OR Nashville OR Memphis OR Texas OR Houston OR Utah OR Vermont OR Virginia OR Seattle OR “West Virginia” OR Wisconsin OR Wyoming OR Washington ouston OR Utah OR Vermont OR Virginia OR Seattle OR "West Virginia" OR Wisconsin OR Wyoming OR Washington) OR SU(“United States” OR USA OR U.S.A. OR U.S. OR Appalachia* OR “Great Lakes” OR mid-Atlantic-state* OR mid-Atlantic-region* OR middle-Atlantic-state* OR middle-Atlantic-region* OR "Midwest* US*" OR "Midwest* U.S*" OR Midwest*-state* OR “Great Plains” OR heartland OR "New England" OR "Northeast* US*" OR "Northeast* U.S*" OR Northeast*-state* OR “Pacific Northwest” OR "northwest* US*" OR "northwest* U.S*" OR Northwest*-state* OR Pacific-state* OR Southeast*-state* OR Southeast*-region OR "Southeast* US*" OR "Southeast* U.S*" OR southern-state* OR "southern US*" OR "southern U.S*" OR "Southwest* state*" OR "Southwest* US*" OR "southwest* U.S*" OR “deep South” OR “Black Belt” OR “Rust Belt” OR “District of Columbia” OR “Washington DC” OR Washington-D.C. OR Alabama OR Alaska OR Arizona OR Arkansas OR "Little Rock" OR California OR “Los Angeles” OR "San Diego" OR "San Francisco" OR

Colorado OR Connecticut OR Delaware OR Florida OR Gainesville OR Jacksonville OR Tampa OR Tallahassee OR Georgia OR Atlanta OR Hawaii OR Hawai'i OR Hawai`i OR Honolulu OR Idaho OR Illinois OR Chicago OR Indiana OR Indianapolis OR "West Lafayette" OR Iowa OR Kansas OR Wichita OR Kentucky OR Louisiana OR “New Orleans” OR “Baton Rouge” OR Shreveport OR Maine OR Orono OR Maryland OR “johns Hopkins” OR Massachusetts OR Boston OR Harvard OR Michigan OR Detroit OR “Ann Arbor” OR “East Lansing” OR Minnesota OR Minneapolis OR Rochester OR Mississippi OR Missouri OR Montana OR Missoula OR Nebraska OR Nevada OR “Las Vegas” OR “New Hampshire” OR “New Jersey” OR “New Mexico” OR “New York” OR “North Carolina” OR “North Dakota” OR Ohio OR Cincinnati OR Oklahoma OR Oregon OR Pennsylvania OR Philadelphia OR “Rhode Island” OR “South Carolina” OR “South Dakota” OR Tennessee OR Nashville OR Memphis OR Texas OR Houston OR Utah OR Vermont OR Virginia OR Seattle OR “West Virginia” OR Wisconsin OR Wyoming OR Washington) **)**

Environmental Complete; EBSCOhost

	**Concept: Community Forests**	**Concept: Health, ecosystem services, economic impacts**
Descriptors<br>DE ()	"Community forests"	"Health"<br>"Rural health"<br>"Public health"<br>"Health impact assessment"<br>"Ecosystem services"<br>"Payments for ecosystem services"<br>"Environmental Economics"<br>"Forest economics"<br>"Economic impact"
Free text terms (searched in Title (TI), Abstract (AB), Keywords (KW) and Term in Subject Heading (SU)	"community forest*"<br>"communal forest*"<br>"community-managed forest*"<br>"social forest*"<br>"local forest*"<br>"commons forest*"<br>"town forest*"<br>"city forest*"<br>"metropolitan forest*"<br>"suburban forest*"<br>"nonurban forest*"<br>"nonmetropolitan forest*"<br>"community woodland*"<br>"communal woodland*"<br>"community-managed woodland*"<br>"social woodland*"<br>"local woodland*"<br>"commons woodland*"<br>"town woodland*"<br>"urban woodland*"<br>"city woodland*"<br>"metropolitan woodland*"<br>"suburban woodland*"<br>"nonurban woodland*"<br>"nonmetropolitan woodland*"<br>"forest commons"	health<br>well-being<br>wellbeing<br>prosper*<br>"economic opportunit*"<br>"economic contribution*"<br>"economic impact*"<br>"economic benefit*"<br>livelihood*<br>"ecosystem service*"

(DE "Community forests" OR TI("community forest*" OR "communal forest*" OR "community-managed forest*" OR "social forest*" OR "local forest*" OR "commons forest*" OR "town forest*" OR "city forest*" OR "metropolitan forest*" OR "suburban forest*" OR "nonurban forest*" OR "nonmetropolitan forest*" OR "community woodland*" OR "communal woodland*" OR "community-managed woodland*" OR "social woodland*" OR "local woodland*" OR "commons woodland*" OR "town woodland*" OR "urban woodland*" OR "city woodland*" OR "metropolitan woodland*" OR "suburban woodland*" OR "nonurban woodland*" OR "nonmetropolitan woodland*" OR "forest commons") OR AB("community forest*" OR "communal forest*" OR "community-managed forest*" OR "social forest*" OR "local forest*" OR "commons forest*" OR "town forest*" OR "city forest*" OR "metropolitan forest*" OR "suburban forest*" OR "nonurban forest*" OR "nonmetropolitan forest*" OR "community woodland*" OR "communal woodland*" OR "community-managed woodland*" OR "social woodland*" OR "local woodland*" OR "commons woodland*" OR

"town woodland*" OR "urban woodland*" OR "city woodland*" OR "metropolitan woodland*" OR "suburban woodland*" OR "nonurban woodland*" OR "nonmetropolitan woodland*" OR "forest commons") OR KW("community forest*" OR "communal forest*" OR "community-managed forest*" OR "social forest*" OR "local forest*" OR "commons forest*" OR "town forest*" OR "city forest*" OR "metropolitan forest*" OR "suburban forest*" OR "nonurban forest*" OR "nonmetropolitan forest*" OR "community woodland*" OR "communal woodland*" OR "community-managed woodland*" OR "social woodland*" OR "local woodland*" OR "commons woodland*" OR "town woodland*" OR "urban woodland*" OR "city woodland*" OR "metropolitan woodland*" OR "suburban woodland*" OR "nonurban woodland*" OR "nonmetropolitan woodland*" OR "forest commons") OR SU("community forest*" OR "communal forest*" OR "community-managed forest*" OR "social forest*" OR "local forest*" OR "commons forest*" OR "town forest*" OR "city forest*" OR "metropolitan forest*" OR "suburban forest*" OR "nonurban forest*" OR "nonmetropolitan forest*" OR "community woodland*" OR "communal woodland*" OR "community-managed woodland*" OR "social woodland*" OR "local woodland*" OR "commons woodland*" OR "town woodland*" OR "urban woodland*" OR "city woodland*" OR "metropolitan woodland*" OR "suburban woodland*" OR "nonurban woodland*" OR "nonmetropolitan woodland*" OR "forest commons") **)**

AND

(DE("Health" OR "Rural health" OR "Public health" OR "Health impact assessment" OR "Ecosystem services" OR "Payments for ecosystem services" OR "Environmental Economics" OR "Forest economics" OR "Economic impact") OR TI(health OR well-being OR wellbeing OR prosper* OR "economic opportunit*" OR "economic contribution*" OR "economic impact*" OR "economic benefit*" OR livelihood* OR "ecosystem service*") OR AB(health OR well-being OR wellbeing OR prosper* OR "economic opportunit*" OR "economic contribution*" OR "economic impact*" OR "economic benefit*" OR livelihood* OR "ecosystem service*") OR KW(health OR well-being OR wellbeing OR prosper* OR "economic opportunit*" OR "economic contribution*" OR "economic impact*" OR "economic benefit*" OR livelihood* OR "ecosystem service*") OR SU(health OR well-being OR wellbeing OR prosper* OR "economic opportunit*" OR "economic contribution*" OR "economic impact*" OR "economic benefit*" OR livelihood* OR "ecosystem service*")**)**
AND

(GE("United States" OR "Appalachian Region" OR "Great Lakes Region" OR "New England" OR "Mid-Atlantic Region" OR "Midwestern United States" OR "Northwestern United States" OR "Pacific States" OR "Southeastern United States" OR "Southwestern United States" OR Alabama OR Alaska OR Arizona OR Arkansas OR California OR Colorado OR Connecticut OR Delaware OR "District of Columbia" OR Florida OR Georgia OR Hawaii OR Idaho OR Illinois OR Indiana OR Iowa OR Kansas OR Kentucky OR Louisiana OR Maine OR Maryland OR Massachusetts OR Michigan OR Minnesota OR Mississippi OR Missouri OR Montana OR Nebraska OR Nevada OR "New Hampshire" OR "New Jersey" OR "New Mexico" OR "New York" OR "North Carolina" OR "North Dakota" OR Ohio OR Oklahoma OR Oregon OR Pennsylvania OR "Rhode Island" OR "South Carolina" OR "South Dakota" OR Tennessee OR Texas OR Utah OR Vermont OR Virginia OR Washington OR "West Virginia" OR Wisconsin OR Wyoming) OR TI("United States" OR USA OR U.S.A. OR U.S. OR Appalachia* OR "Great Lakes" OR mid-Atlantic-state* OR mid-Atlantic-region* OR middle-Atlantic-state* OR middle-Atlantic-region* OR "Midwest* US*" OR "Midwest* U.S*" OR Midwest*-state* OR "Great Plains" OR heartland OR "New England" OR "Northeast* US*" OR "Northeast* U.S*" OR Northeast*-state* OR "Pacific Northwest" OR "northwest* US*" OR "northwest* U.S*" OR Northwest*-state* OR Pacific-state* OR Southeast*-state* OR Southeast*-region OR "Southeast* US*" OR "Southeast* U.S*" OR southern-state* OR "southern US*" OR "southern U.S*" OR "Southwest* state*" OR "Southwest* US*" OR "southwest* U.S*" OR "deep South" OR "Black Belt" OR "Rust Belt" OR "District of Columbia" OR "Washington DC" OR Washington-D.C. OR Alabama OR Alaska OR Arizona OR Arkansas OR "Little Rock" OR California OR "Los Angeles" OR "San Diego" OR "San Francisco" OR Colorado OR Connecticut OR Delaware OR Florida OR Gainesville OR Jacksonville OR Tampa OR Tallahassee OR Georgia OR Atlanta OR Hawaii OR Hawai'i OR Hawai`i OR Honolulu OR Idaho OR Illinois OR Chicago OR Indiana OR Indianapolis OR "West Lafayette" OR Iowa OR Kansas OR Wichita OR Kentucky OR Louisiana OR "New Orleans" OR "Baton Rouge" OR Shreveport OR Maine OR Orono OR Maryland OR "johns Hopkins" OR Massachusetts OR Boston OR Harvard OR Michigan OR Detroit OR "Ann Arbor" OR "East Lansing" OR Minnesota OR Minneapolis OR Rochester OR Mississippi OR Missouri OR Montana OR Missoula OR Nebraska OR Nevada OR "Las Vegas" OR "New Hampshire" OR "New Jersey" OR "New Mexico" OR "New York" OR

“North Carolina” OR “North Dakota” OR Ohio OR Cincinnati OR Oklahoma OR Oregon OR Pennsylvania OR Philadelphia OR “Rhode Island” OR “South Carolina” OR “South Dakota” OR Tennessee OR Nashville OR Memphis OR Texas OR Houston OR Utah OR Vermont OR Virginia OR Seattle OR “West Virginia” OR Wisconsin OR Wyoming OR Washington) OR AB("“United States” OR USA OR U.S.A. OR U.S. OR Appalachia* OR “Great Lakes” OR mid-Atlantic-state* OR mid-Atlantic-region* OR middle-Atlantic-state* OR middle-Atlantic-region* OR "Midwest* US*" OR "Midwest* U.S*" OR Midwest*-state* OR “Great Plains” OR heartland OR "New England" OR "Northeast* US*" OR "Northeast* U.S*" OR Northeast*-state* OR “Pacific Northwest” OR "northwest* US*" OR "northwest* U.S*" OR Northwest*-state* OR Pacific-state* OR Southeast*-state* OR Southeast*-region OR "Southeast* US*" OR "Southeast* U.S*" OR southern-state* OR "southern US*" OR "southern U.S*" OR "Southwest* state*" OR "Southwest* US*" OR "southwest* U.S*" OR “deep South” OR “Black Belt” OR “Rust Belt” OR “District of Columbia” OR “Washington DC” OR Washington-D.C. OR Alabama OR Alaska OR Arizona OR Arkansas OR "Little Rock" OR California OR “Los Angeles” OR "San Diego" OR "San Francisco" OR Colorado OR Connecticut OR Delaware OR Florida OR Gainesville OR Jacksonville OR Tampa OR Tallahassee OR Georgia OR Atlanta OR Hawaii OR Hawai'i OR Hawai`i OR Honolulu OR Idaho OR Illinois OR Chicago OR Indiana OR Indianapolis OR "West Lafayette" OR Iowa OR Kansas OR Wichita OR Kentucky OR Louisiana OR “New Orleans” OR “Baton Rouge” OR Shreveport OR Maine OR Orono OR Maryland OR “johns Hopkins” OR Massachusetts OR Boston OR Harvard OR Michigan OR Detroit OR “Ann Arbor” OR “East Lansing” OR Minnesota OR Minneapolis OR Rochester OR Mississippi OR Missouri OR Montana OR Missoula OR Nebraska OR Nevada OR “Las Vegas” OR “New Hampshire” OR “New Jersey” OR “New Mexico” OR “New York” OR “North Carolina” OR “North Dakota” OR Ohio OR Cincinnati OR Oklahoma OR Oregon OR Pennsylvania OR Philadelphia OR “Rhode Island” OR “South Carolina” OR “South Dakota” OR Tennessee OR Nashville OR Memphis OR Texas OR Houston OR Utah OR Vermont OR Virginia OR Seattle OR “West Virginia” OR Wisconsin OR Wyoming OR Washington) OR KW(“United States” OR USA OR U.S.A. OR U.S. OR Appalachia* OR “Great Lakes” OR mid-Atlantic-state* OR mid-Atlantic-region* OR middle-Atlantic-state* OR middle-Atlantic-region* OR "Midwest* US*" OR "Midwest* U.S*" OR Midwest*-state* OR “Great Plains” OR heartland OR "New England" OR "Northeast* US*" OR "Northeast* U.S*" OR Northeast*-state* OR “Pacific Northwest” OR "northwest* US*" OR "northwest* U.S*" OR Northwest*-state* OR Pacific-state* OR Southeast*-state* OR Southeast*-region OR "Southeast* US*" OR "Southeast* U.S*" OR southern-state* OR "southern US*" OR "southern U.S*" OR "Southwest* state*" OR "Southwest* US*" OR "southwest* U.S*" OR “deep South” OR “Black Belt” OR “Rust Belt” OR “District of Columbia” OR “Washington DC” OR Washington-D.C. OR Alabama OR Alaska OR Arizona OR Arkansas OR "Little Rock" OR California OR “Los Angeles” OR "San Diego" OR "San Francisco" OR Colorado OR Connecticut OR Delaware OR Florida OR Gainesville OR Jacksonville OR Tampa OR Tallahassee OR Georgia OR Atlanta OR Hawaii OR Hawai'i OR Hawai`i OR Honolulu OR Idaho OR Illinois OR Chicago OR Indiana OR Indianapolis OR "West Lafayette" OR Iowa OR Kansas OR Wichita OR Kentucky OR Louisiana OR “New Orleans” OR “Baton Rouge” OR Shreveport OR Maine OR Orono OR Maryland OR “johns Hopkins” OR Massachusetts OR Boston OR Harvard OR Michigan OR Detroit OR “Ann Arbor” OR “East Lansing” OR Minnesota OR Minneapolis OR Rochester OR Mississippi OR Missouri OR Montana OR Missoula OR Nebraska OR Nevada OR “Las Vegas” OR “New Hampshire” OR “New Jersey” OR “New Mexico” OR “New York” OR “North Carolina” OR “North Dakota” OR Ohio OR Cincinnati OR Oklahoma OR Oregon OR Pennsylvania OR Philadelphia OR “Rhode Island” OR “South Carolina” OR “South Dakota” OR Tennessee OR Nashville OR Memphis OR Texas OR Houston OR Utah OR Vermont OR Virginia OR Seattle OR “West Virginia” OR Wisconsin OR Wyoming OR Washington) OR SU(“United States” OR USA OR U.S.A. OR U.S. OR Appalachia* OR “Great Lakes” OR mid-Atlantic-state* OR mid-Atlantic-region* OR middle-Atlantic-state* OR middle-Atlantic-region* OR "Midwest* US*" OR "Midwest* U.S*" OR Midwest*-state* OR “Great Plains” OR heartland OR "New England" OR "Northeast* US*" OR "Northeast* U.S*" OR Northeast*-state* OR “Pacific Northwest” OR "northwest* US*" OR "northwest* U.S*" OR Northwest*-state* OR Pacific-state* OR Southeast*-state* OR Southeast*-region OR "Southeast* US*" OR "Southeast* U.S*" OR southern-state* OR "southern US*" OR "southern U.S*" OR "Southwest* state*" OR "Southwest* US*" OR "southwest* U.S*" OR “deep South” OR “Black Belt” OR “Rust Belt” OR “District of Columbia” OR “Washington DC” OR Washington-D.C. OR Alabama OR Alaska OR Arizona OR Arkansas OR "Little Rock" OR California OR “Los Angeles” OR "San Diego" OR "San Francisco" OR Colorado OR Connecticut OR Delaware OR Florida OR Gainesville OR Jacksonville OR Tampa OR Tallahassee OR Georgia OR Atlanta OR Hawaii OR Hawai'i OR Hawai`i OR Honolulu OR Idaho OR Illinois OR Chicago OR Indiana OR Indianapolis OR "West Lafayette" OR Iowa OR Kansas OR Wichita OR Kentucky OR Louisiana OR “New

Orleans” OR “Baton Rouge” OR Shreveport OR Maine OR Orono OR Maryland OR “johns Hopkins” OR Massachusetts OR Boston OR Harvard OR Michigan OR Detroit OR “Ann Arbor” OR “East Lansing” OR Minnesota OR Minneapolis OR Rochester OR Mississippi OR Missouri OR Montana OR Missoula OR Nebraska OR Nevada OR “Las Vegas” OR “New Hampshire” OR “New Jersey” OR “New Mexico” OR “New York” OR “North Carolina” OR “North Dakota” OR Ohio OR Cincinnati OR Oklahoma OR Oregon OR Pennsylvania OR Philadelphia OR “Rhode Island” OR “South Carolina” OR “South Dakota” OR Tennessee OR Nashville OR Memphis OR Texas OR Houston OR Utah OR Vermont OR Virginia OR Seattle OR “West Virginia” OR Wisconsin OR Wyoming OR Washington))

Running note: On Advanced Search screen, make sure the following boxes are not checked: "Apply related words", "Also search within the full text of the articles", "Apply equivalent subjects".

To limit by date, enter your date range on the Advanced Search screen or use the slider in the results sidebar.

To limit by publication type, select all relevant types in the Source Types options in the results sidebar.

GreenFILE

	Concept: Community Forests	Concept: Health, ecosystem services, economic impacts
Descriptors<br>DE ()	"Cooperative Forests & Forestry"<br>"Community Forests"<br>"County Forests"<br>"Community Forestry"	"Health"<br>"Rural Health"<br>"Infrastructure (Economics)"<br>"Land Economics"<br>"Ecological Economics"<br>"Environmental Protection & Economics"<br>"Environmental Economics"<br>"Land Use & The Environment"<br>"Payments for Ecosystem Services"
Free text terms (searched in Title (TI), Abstract (AB), Keywords (KW) and Term in Subject Heading (SU)	"community forest*"<br>"communal forest*"<br>"community-managed forest*"<br>"social forest*"<br>"local forest*"<br>"commons forest*"<br>"town forest*"<br>"city forest*"<br>"metropolitan forest*"<br>"suburban forest*"<br>"nonurban forest*"<br>"nonmetropolitan forest*"<br>"community woodland*"<br>"communal woodland*"<br>"community-managed woodland*"<br>"social woodland*"<br>"local woodland*"<br>"commons woodland*"<br>"town woodland*"<br>"urban woodland*"<br>"city woodland*"<br>"metropolitan woodland*"<br>"suburban woodland*"<br>"nonurban woodland*"<br>"nonmetropolitan woodland*"<br>"forest commons"	health<br>well-being<br>wellbeing<br>prosper*<br>"economic opportunit*"<br>"economic contribution*"<br>"economic impact*"<br>"economic benefit*"<br>livelihood*<br>"ecosystem service*"

(DE("Cooperative Forests & Forestry" OR "Community Forests" OR "County Forests" OR "Community Forestry") OR TI("community forest*" OR "communal forest*" OR "community-managed forest*" OR "social forest*" OR "local forest*" OR "commons forest*" OR "town forest*" OR "city forest*" OR "metropolitan forest*" OR "suburban forest*" OR "nonurban forest*" OR "nonmetropolitan forest*" OR "community woodland*" OR "communal woodland*" OR "community-managed woodland*" OR "social woodland*" OR "local woodland*" OR "commons woodland*" OR "town woodland*" OR "urban woodland*" OR "city woodland*" OR "metropolitan woodland*" OR "suburban woodland*" OR "nonurban woodland*" OR "nonmetropolitan woodland*" OR "forest commons") OR AB("community forest*" OR "communal forest*" OR "community-managed forest*" OR "social forest*" OR "local forest*" OR "commons forest*" OR "town forest*" OR "city forest*" OR "metropolitan forest*" OR "suburban forest*" OR "nonurban forest*" OR "nonmetropolitan forest*" OR "community woodland*" OR "communal woodland*" OR "community-managed woodland*" OR "social woodland*" OR "local woodland*" OR "commons woodland*" OR "town woodland*" OR "urban

woodland*" OR "city woodland*" OR "metropolitan woodland*" OR "suburban woodland*" OR "nonurban woodland*" OR "nonmetropolitan woodland*" OR "forest commons") OR KW("community forest*" OR "communal forest*" OR "community-managed forest*" OR "social forest*" OR "local forest*" OR "commons forest*" OR "town forest*" OR "city forest*" OR "metropolitan forest*" OR "suburban forest*" OR "nonurban forest*" OR "nonmetropolitan forest*" OR "community woodland*" OR "communal woodland*" OR "community-managed woodland*" OR "social woodland*" OR "local woodland*" OR "commons woodland*" OR "town woodland*" OR "urban woodland*" OR "city woodland*" OR "metropolitan woodland*" OR "suburban woodland*" OR "nonurban woodland*" OR "nonmetropolitan woodland*" OR "forest commons") OR SU("community forest*" OR "communal forest*" OR "community-managed forest*" OR "social forest*" OR "local forest*" OR "commons forest*" OR "town forest*" OR "city forest*" OR "metropolitan forest*" OR "suburban forest*" OR "nonurban forest*" OR "nonmetropolitan forest*" OR "community woodland*" OR "communal woodland*" OR "community-managed woodland*" OR "social woodland*" OR "local woodland*" OR "commons woodland*" OR "town woodland*" OR "urban woodland*" OR "city woodland*" OR "metropolitan woodland*" OR "suburban woodland*" OR "nonurban woodland*" OR "nonmetropolitan woodland*" OR "forest commons") **)**

AND

(DE("Health" OR "Rural Health" OR "Infrastructure (Economics)" OR "Land Economics" OR "Ecological Economics" OR "Environmental Protection & Economics" OR "Environmental Economics" OR "Land Use & The Environment" OR "Payments for Ecosystem Services") OR TI(health OR well-being OR wellbeing OR prosper* OR "economic opportunit*" OR "economic contribution*" OR "economic impact*" OR "economic benefit*" OR livelihood* OR "ecosystem service*") OR AB(health OR well-being OR wellbeing OR prosper* OR "economic opportunit*" OR "economic contribution*" OR "economic impact*" OR "economic benefit*" OR livelihood* OR "ecosystem service*") OR KW(health OR well-being OR wellbeing OR prosper* OR "economic opportunit*" OR "economic contribution*" OR "economic impact*" OR "economic benefit*" OR livelihood* OR "ecosystem service*") OR SU(health OR well-being OR wellbeing OR prosper* OR "economic opportunit*" OR "economic contribution*" OR "economic impact*" OR "economic benefit*" OR livelihood* OR "ecosystem service*")**)**

AND

(ZG("United States" OR "Appalachian Region" OR "Great Lakes Region" OR "New England" OR "Mid-Atlantic Region" OR "Midwestern United States" OR "Northwestern United States" OR "Pacific States" OR "Southeastern United States" OR "Southwestern United States" OR Alabama OR Alaska OR Arizona OR Arkansas OR California OR Colorado OR Connecticut OR Delaware OR "District of Columbia" OR Florida OR Georgia OR Hawaii OR Idaho OR Illinois OR Indiana OR Iowa OR Kansas OR Kentucky OR Louisiana OR Maine OR Maryland OR Massachusetts OR Michigan OR Minnesota OR Mississippi OR Missouri OR Montana OR Nebraska OR Nevada OR "New Hampshire" OR "New Jersey" OR "New Mexico" OR "New York" OR "North Carolina" OR "North Dakota" OR Ohio OR Oklahoma OR Oregon OR Pennsylvania OR "Rhode Island" OR "South Carolina" OR "South Dakota" OR Tennessee OR Texas OR Utah OR Vermont OR Virginia OR Washington OR "West Virginia" OR Wisconsin OR Wyoming) OR TI(“United States” OR USA OR U.S.A. OR U.S. OR Appalachia* OR “Great Lakes” OR mid-Atlantic-state* OR mid-Atlantic-region* OR middle-Atlantic-state* OR middle-Atlantic-region* OR "Midwest* US*" OR "Midwest* U.S*" OR Midwest*-state* OR “Great Plains” OR heartland OR "New England" OR "Northeast* US*" OR "Northeast* U.S*" OR Northeast*-state* OR “Pacific Northwest” OR "northwest* US*" OR "northwest* U.S*" OR Northwest*-state* OR Pacific-state* OR Southeast*-state* OR Southeast*-region OR "Southeast* US*" OR "Southeast* U.S*" OR southern-state* OR "southern US*" OR "southern U.S*" OR "Southwest* state*" OR "Southwest* US*" OR "southwest* U.S*" OR “deep South” OR “Black Belt” OR “Rust Belt” OR “District of Columbia” OR “Washington DC” OR Washington-D.C. OR Alabama OR Alaska OR Arizona OR Arkansas OR "Little Rock" OR California OR “Los Angeles” OR "San Diego" OR "San Francisco" OR Colorado OR Connecticut OR Delaware OR Florida OR Gainesville OR Jacksonville OR Tampa OR Tallahassee OR Georgia OR Atlanta OR Hawaii OR Hawai'i OR Hawai`i OR Honolulu OR Idaho OR Illinois OR Chicago OR Indiana OR Indianapolis OR "West Lafayette" OR Iowa OR Kansas OR Wichita OR Kentucky OR Louisiana OR “New Orleans” OR “Baton Rouge” OR Shreveport OR Maine OR Orono OR Maryland OR “johns Hopkins” OR Massachusetts OR Boston OR Harvard OR Michigan OR Detroit OR “Ann Arbor” OR “East Lansing” OR Minnesota OR Minneapolis OR Rochester OR Mississippi OR Missouri OR Montana OR Missoula OR Nebraska OR Nevada OR “Las Vegas” OR “New Hampshire” OR “New Jersey” OR “New Mexico” OR “New York” OR “North Carolina” OR “North Dakota” OR Ohio OR Cincinnati OR Oklahoma OR Oregon OR Pennsylvania OR Philadelphia

OR “Rhode Island” OR “South Carolina” OR “South Dakota” OR Tennessee OR Nashville OR Memphis OR Texas OR Houston OR Utah OR Vermont OR Virginia OR Seattle OR “West Virginia” OR Wisconsin OR Wyoming OR Washington) OR AB(“United States” OR USA OR U.S.A. OR U.S. OR Appalachia* OR “Great Lakes” OR mid-Atlantic-state* OR mid-Atlantic-region* OR middle-Atlantic-state* OR middle-Atlantic-region* OR "Midwest* US*" OR "Midwest* U.S*" OR Midwest*-state* OR “Great Plains” OR heartland OR "New England" OR "Northeast* US*" OR "Northeast* U.S*" OR Northeast*-state* OR “Pacific Northwest” OR "northwest* US*" OR "northwest* U.S*" OR Northwest*-state* OR Pacific-state* OR Southeast*-state* OR Southeast*-region OR "Southeast* US*" OR "Southeast* U.S*" OR southern-state* OR "southern US*" OR "southern U.S*" OR "Southwest* state*" OR "Southwest* US*" OR "southwest* U.S*" OR “deep South” OR “Black Belt” OR “Rust Belt” OR “District of Columbia” OR “Washington DC” OR Washington-D.C. OR Alabama OR Alaska OR Arizona OR Arkansas OR "Little Rock" OR California OR “Los Angeles” OR "San Diego" OR "San Francisco" OR Colorado OR Connecticut OR Delaware OR Florida OR Gainesville OR Jacksonville OR Tampa OR Tallahassee OR Georgia OR Atlanta OR Hawaii OR Hawai'i OR Hawai`i OR Honolulu OR Idaho OR Illinois OR Chicago OR Indiana OR Indianapolis OR "West Lafayette" OR Iowa OR Kansas OR Wichita OR Kentucky OR Louisiana OR “New Orleans” OR “Baton Rouge” OR Shreveport OR Maine OR Orono OR Maryland OR “johns Hopkins” OR Massachusetts OR Boston OR Harvard OR Michigan OR Detroit OR “Ann Arbor” OR “East Lansing” OR Minnesota OR Minneapolis OR Rochester OR Mississippi OR Missouri OR Montana OR Missoula OR Nebraska OR Nevada OR “Las Vegas” OR “New Hampshire” OR “New Jersey” OR “New Mexico” OR “New York” OR “North Carolina” OR “North Dakota” OR Ohio OR Cincinnati OR Oklahoma OR Oregon OR Pennsylvania OR Philadelphia OR “Rhode Island” OR “South Carolina” OR “South Dakota” OR Tennessee OR Nashville OR Memphis OR Texas OR Houston OR Utah OR Vermont OR Virginia OR Seattle OR “West Virginia” OR Wisconsin OR Wyoming OR Washington) OR KW(“United States” OR USA OR U.S.A. OR U.S. OR Appalachia* OR “Great Lakes” OR mid-Atlantic-state* OR mid-Atlantic-region* OR middle-Atlantic-state* OR middle-Atlantic-region* OR "Midwest* US*" OR "Midwest* U.S*" OR Midwest*-state* OR “Great Plains” OR heartland OR "New England" OR "Northeast* US*" OR "Northeast* U.S*" OR Northeast*-state* OR “Pacific Northwest” OR "northwest* US*" OR "northwest* U.S*" OR Northwest*-state* OR Pacific-state* OR Southeast*-state* OR Southeast*-region OR "Southeast* US*" OR "Southeast* U.S*" OR southern-state* OR "southern US*" OR "southern U.S*" OR "Southwest* state*" OR "Southwest* US*" OR "southwest* U.S*" OR “deep South” OR “Black Belt” OR “Rust Belt” OR “District of Columbia” OR “Washington DC” OR Washington-D.C. OR Alabama OR Alaska OR Arizona OR Arkansas OR "Little Rock" OR California OR “Los Angeles” OR "San Diego" OR "San Francisco" OR Colorado OR Connecticut OR Delaware OR Florida OR Gainesville OR Jacksonville OR Tampa OR Tallahassee OR Georgia OR Atlanta OR Hawaii OR Hawai'i OR Hawai`i OR Honolulu OR Idaho OR Illinois OR Chicago OR Indiana OR Indianapolis OR "West Lafayette" OR Iowa OR Kansas OR Wichita OR Kentucky OR Louisiana OR “New Orleans” OR “Baton Rouge” OR Shreveport OR Maine OR Orono OR Maryland OR “johns Hopkins” OR Massachusetts OR Boston OR Harvard OR Michigan OR Detroit OR “Ann Arbor” OR “East Lansing” OR Minnesota OR Minneapolis OR Rochester OR Mississippi OR Missouri OR Montana OR Missoula OR Nebraska OR Nevada OR “Las Vegas” OR “New Hampshire” OR “New Jersey” OR “New Mexico” OR “New York” OR “North Carolina” OR “North Dakota” OR Ohio OR Cincinnati OR Oklahoma OR Oregon OR Pennsylvania OR Philadelphia OR “Rhode Island” OR “South Carolina” OR “South Dakota” OR Tennessee OR Nashville OR Memphis OR Texas OR Houston OR Utah OR Vermont OR Virginia OR Seattle OR “West Virginia” OR Wisconsin OR Wyoming OR Washington) OR SU(“United States” OR USA OR U.S.A. OR U.S. OR Appalachia* OR “Great Lakes” OR mid-Atlantic-state* OR mid-Atlantic-region* OR middle-Atlantic-state* OR middle-Atlantic-region* OR "Midwest* US*" OR "Midwest* U.S*" OR Midwest*-state* OR “Great Plains” OR heartland OR "New England" OR "Northeast* US*" OR "Northeast* U.S*" OR Northeast*-state* OR “Pacific Northwest” OR "northwest* US*" OR "northwest* U.S*" OR Northwest*-state* OR Pacific-state* OR Southeast*-state* OR Southeast*-region OR "Southeast* US*" OR "Southeast* U.S*" OR southern-state* OR "southern US*" OR "southern U.S*" OR "Southwest* state*" OR "Southwest* US*" OR "southwest* U.S*" OR “deep South” OR “Black Belt” OR “Rust Belt” OR “District of Columbia” OR “Washington DC” OR Washington-D.C. OR Alabama OR Alaska OR Arizona OR Arkansas OR "Little Rock" OR California OR “Los Angeles” OR "San Diego" OR "San Francisco" OR Colorado OR Connecticut OR Delaware OR Florida OR Gainesville OR Jacksonville OR Tampa OR Tallahassee OR Georgia OR Atlanta OR Hawaii OR Hawai'i OR Hawai`i OR Honolulu OR Idaho OR Illinois OR Chicago OR Indiana OR Indianapolis OR "West Lafayette" OR Iowa OR Kansas OR Wichita OR Kentucky OR Louisiana OR “New Orleans” OR “Baton Rouge” OR Shreveport OR Maine OR Orono OR Maryland OR “johns Hopkins” OR Massachusetts OR

Boston OR Harvard OR Michigan OR Detroit OR “Ann Arbor” OR “East Lansing” OR Minnesota OR Minneapolis OR Rochester OR Mississippi OR Missouri OR Montana OR Missoula OR Nebraska OR Nevada OR “Las Vegas” OR “New Hampshire” OR “New Jersey” OR “New Mexico” OR “New York” OR “North Carolina” OR “North Dakota” OR Ohio OR Cincinnati OR Oklahoma OR Oregon OR Pennsylvania OR Philadelphia OR “Rhode Island” OR “South Carolina” OR “South Dakota” OR Tennessee OR Nashville OR Memphis OR Texas OR Houston OR Utah OR Vermont OR Virginia OR Seattle OR “West Virginia” OR Wisconsin OR Wyoming OR Washington))

ProQuest Agricultural & Environmental Science Collection

	Concept: Community Forests	Concept: Health, ecosystem services, economic impacts
Subject headings (SUBJECT.EXACT)	SUBJECT.EXACT("Community facilities" OR "Community involvement" OR "Community organizations")<br>AND<br>SUBJECT.EXACT("Forests" OR "Boreal forests" OR "Cloud forests" OR "Coniferous forests" OR "Deciduous forests" OR "Dry forests" OR "Mixed forests" OR "Mountain forests" OR "National forests" OR "Old growth" OR "Rainforests" OR "Riparian forests" OR "Temperate forests" OR "Woodlands")	"Health"<br>"Environmental health"<br>"Economics"<br>"Environmental economics"<br>"Economic impact"<br>"Economic importance"<br>"Ecosystem services"
Free text terms	"community forest*"<br>"communal forest*"<br>"community-managed forest*"<br>"social forest*"<br>"local forest*"<br>"commons forest*"<br>"town forest*"<br>"city forest*"<br>"metropolitan forest*"<br>"suburban forest*"<br>"nonurban forest*"<br>"nonmetropolitan forest*"<br>"community woodland*"<br>"communal woodland*"<br>"community-managed woodland*"<br>"social woodland*"<br>"local woodland*"<br>"commons woodland*"<br>"town woodland*"<br>"urban woodland*"<br>"city woodland*"<br>"metropolitan woodland*"<br>"suburban woodland*"<br>"nonurban woodland*"<br>"nonmetropolitan woodland*"<br>"forest commons"	health<br>well-being<br>wellbeing<br>prosper*<br>"economic opportunit*"<br>"economic contribution*"<br>"economic impact*"<br>"economic benefit*"<br>livelihood*<br>"ecosystem service*"

((SUBJECT.EXACT("Community facilities" OR "Community involvement" OR "Community organizations") AND SUBJECT.EXACT("Forests" OR "Boreal forests" OR "Cloud forests" OR "Coniferous forests" OR "Deciduous forests" OR "Dry forests" OR "Mixed forests" OR "Mountain forests" OR "National forests" OR "Old growth" OR "Rainforests" OR "Riparian forests" OR "Temperate forests" OR "Woodlands")) OR title("community forest*" OR "communal forest*" OR "community-managed forest*" OR "social forest*" OR "local forest*" OR "commons forest*" OR "town forest*" OR "city forest*" OR "metropolitan forest*" OR "suburban forest*" OR "nonurban forest*" OR "nonmetropolitan forest*" OR "community woodland*" OR "communal woodland*" OR "community-managed woodland*" OR "social woodland*" OR "local woodland*" OR "commons woodland*" OR "town woodland*" OR "urban woodland*" OR "city woodland*" OR

"metropolitan woodland*" OR "suburban woodland*" OR "nonurban woodland*" OR "nonmetropolitan woodland*" OR "forest commons") OR summary("community forest*" OR "communal forest*" OR "community-managed forest*" OR "social forest*" OR "local forest*" OR "commons forest*" OR "town forest*" OR "city forest*" OR "metropolitan forest*" OR "suburban forest*" OR "nonurban forest*" OR "nonmetropolitan forest*" OR "community woodland*" OR "communal woodland*" OR "community-managed woodland*" OR "social woodland*" OR "local woodland*" OR "commons woodland*" OR "town woodland*" OR "urban woodland*" OR "city woodland*" OR "metropolitan woodland*" OR "suburban woodland*" OR "nonurban woodland*" OR "nonmetropolitan woodland*" OR "forest commons") OR subject("community forest*" OR "communal forest*" OR "community-managed forest*" OR "social forest*" OR "local forest*" OR "commons forest*" OR "town forest*" OR "city forest*" OR "metropolitan forest*" OR "suburban forest*" OR "nonurban forest*" OR "nonmetropolitan forest*" OR "community woodland*" OR "communal woodland*" OR "community-managed woodland*" OR "social woodland*" OR "local woodland*" OR "commons woodland*" OR "town woodland*" OR "urban woodland*" OR "city woodland*" OR "metropolitan woodland*" OR "suburban woodland*" OR "nonurban woodland*" OR "nonmetropolitan woodland*" OR "forest commons")**)**

AND

(SUBJECT.EXACT("Health" OR "Environmental health" OR "Economics" OR "Environmental economics" OR "Economic impact" OR "Economic importance" OR "Ecosystem services") OR title(health OR well-being OR wellbeing OR prosper* OR "economic opportunit*" OR "economic contribution*" OR "economic impact*" OR "economic benefit*" OR livelihood* OR "ecosystem service*") OR summary(health OR well-being OR wellbeing OR prosper* OR "economic opportunit*" OR "economic contribution*" OR "economic impact*" OR "economic benefit*" OR livelihood* OR "ecosystem service*") OR subject(health OR well-being OR wellbeing OR prosper* OR "economic opportunit*" OR "economic contribution*" OR "economic impact*" OR "economic benefit*" OR livelihood* OR "ecosystem service*")**)**

AND

(LOCATION("United States" OR "Appalachian Region" OR "Great Lakes Region" OR "New England" OR "Mid-Atlantic Region" OR "Midwestern United States" OR "Northwestern United States" OR "Pacific States" OR "Southeastern United States" OR "Southwestern United States" OR Alabama OR Alaska OR Arizona OR Arkansas OR California OR Colorado OR Connecticut OR Delaware OR "District of Columbia" OR Florida OR Georgia OR Hawaii OR Idaho OR Illinois OR Indiana OR Iowa OR Kansas OR Kentucky OR Louisiana OR Maine OR Maryland OR Massachusetts OR Michigan OR Minnesota OR Mississippi OR Missouri OR Montana OR Nebraska OR Nevada OR "New Hampshire" OR "New Jersey" OR "New Mexico" OR "New York" OR "North Carolina" OR "North Dakota" OR Ohio OR Oklahoma OR Oregon OR Pennsylvania OR "Rhode Island" OR "South Carolina" OR "South Dakota" OR Tennessee OR Texas OR Utah OR Vermont OR Virginia OR Washington OR "West Virginia" OR Wisconsin OR Wyoming) OR title("United States" OR USA OR U.S.A. OR U.S. OR Appalachia* OR "Great Lakes" OR mid-Atlantic-state* OR mid-Atlantic-region* OR middle-Atlantic-state* OR middle-Atlantic-region* OR midwestern-US* OR midwestern-U.S* OR midwestern-state* OR Midwest-state* OR Midwest-US* OR Midwest-U.S* OR "Great Plains" OR heartland OR "New England" OR northeastern-US* OR northeastern-U.S* OR northeastern-state* OR northeast-state* OR northeast-US* OR northeast-U.S* OR "Pacific Northwest" OR northwestern-US* OR northwestern-U.S* OR northwest-U.S* OR northwest-US* OR northwestern-state* OR northwest-state* OR Pacific-state* OR southeast-state* OR southeastern-state* OR southeast-region OR southeastern-region OR southeast-US* OR southeastern-US* OR southeast-U.S* OR southeastern-U.S* OR southern-state* OR southern-US* OR southern-U.S* OR southwest-state* OR southwestern-state* OR southwest-US* OR southwestern-US* OR southwest-U.S* OR southwestern-U.S* OR "deep South" OR "Black Belt" OR "Rust Belt" OR "District of Columbia" OR "Washington DC" OR Washington-D.C. OR Alabama OR Alaska OR Arizona OR Arkansas OR "Little Rock" OR California OR "Los Angeles" OR "San Diego" OR "San Francisco" OR Colorado OR Connecticut OR Delaware OR Florida OR Gainesville OR Jacksonville OR Tampa OR Tallahassee OR Georgia OR Atlanta OR Hawai* OR Honolulu OR Idaho OR Illinois OR Chicago OR Indiana OR Indianapolis OR "West Lafayette" OR Iowa OR Kansas OR Wichita OR Kentucky OR Louisiana OR "New Orleans" OR "Baton Rouge" OR Shreveport OR Maine OR Orono OR Maryland OR Massachusetts OR Boston OR Harvard OR Michigan OR Detroit OR "Ann Arbor" OR "East Lansing" OR Minnesota OR Minneapolis OR Rochester OR Mississippi OR Missouri OR Montana OR Missoula OR Nebraska OR Nevada OR "Las Vegas" OR "New Hampshire" OR "New Jersey" OR "New

Mexico" OR "New York" OR "North Carolina" OR "North Dakota" OR Ohio OR Cincinnati OR Oklahoma OR Oregon OR Pennsylvania OR Philadelphia OR "Rhode Island" OR "South Carolina" OR "South Dakota" OR Tennessee OR Nashville OR Memphis OR Texas OR Houston OR Utah OR Vermont OR Virginia OR Seattle OR "West Virginia" OR Wisconsin OR Wyoming OR Washington) OR abstract("United States" OR USA OR U.S.A. OR U.S. OR Appalachia* OR "Great Lakes" OR mid-Atlantic-state* OR mid-Atlantic-region* OR middle-Atlantic-state* OR middle-Atlantic-region* OR midwestern-US* OR midwestern-U.S* OR midwestern-state* OR Midwest-state* OR Midwest-US* OR Midwest-U.S* OR "Great Plains" OR heartland OR "New England" OR northeastern-US OR northeastern-U.S. OR northeastern-USA OR northeastern-U.S.A. OR northeastern-state* OR northeast-state* OR northeast-US OR northeast-U.S. OR northeast-USA OR northeast-U.S.A. OR "Pacific Northwest" OR northwestern-US OR northwestern-U.S OR northwest-U.S OR northwest-US OR northwestern-USA OR northwestern-U.S.A. OR northwest-U.S.A. OR northwest-USA OR northwestern-state* OR northwest-state* OR Pacific-state* OR southeast-state* OR southeastern-state* OR southeast-region OR southeastern-region OR southeast-US OR southeastern-US OR southeast-U.S OR southeastern-U.S OR southeast-USA OR southeastern-USA OR southeast-U.S.A. OR southeastern-U.S.A. OR southern-state* OR southern-US OR southern-U.S OR southern-USA OR southern-U.S.A. OR southwest-state* OR southwestern-state* OR southwest-US OR southwestern-US OR southwest-U.S OR southwestern-U.S OR southwest-USA OR southwestern-USA OR southwest-U.S.A. OR southwestern-U.S.A. OR "deep South" OR "Black Belt" OR "Rust Belt" OR "District of Columbia" OR "Washington DC" OR Washington-D.C. OR Alabama OR Alaska OR Arizona OR Arkansas OR "Little Rock" OR California OR "Los Angeles" OR "San Diego" OR "San Francisco" OR Colorado OR Connecticut OR Delaware OR Florida OR Gainesville OR Jacksonville OR Tampa OR Tallahassee OR Georgia OR Atlanta OR Hawai* OR Honolulu OR Idaho OR Illinois OR Chicago OR Indiana OR Indianapolis OR "West Lafayette" OR Iowa OR Kansas OR Wichita OR Kentucky OR Louisiana OR "New Orleans" OR "Baton Rouge" OR Shreveport OR Maine OR Orono OR Maryland OR Massachusetts OR Boston OR Harvard OR Michigan OR Detroit OR "Ann Arbor" OR "East Lansing" OR Minnesota OR Minneapolis OR Rochester OR Mississippi OR Missouri OR Montana OR Missoula OR Nebraska OR Nevada OR "Las Vegas" OR "New Hampshire" OR "New Jersey" OR "New Mexico" OR "New York" OR "North Carolina" OR "North Dakota" OR Ohio OR Cincinnati OR Oklahoma OR Oregon OR Pennsylvania OR Philadelphia OR "Rhode Island" OR "South Carolina" OR "South Dakota" OR Tennessee OR Nashville OR Memphis OR Texas OR Houston OR Utah OR Vermont OR Virginia OR Seattle OR "West Virginia" OR Wisconsin OR Wyoming OR Washington))

Web of Science Core Collection – SCI-EXPANDED, SSCI, AHCI, ESCI

	Concept: Community Forests	Concept: Health, ecosystem services, economic impacts
Topic<br>(searches title, abstract, author keywords, Keywords Plus®)	"community forest*"<br>"communal forest*"<br>"community-managed forest*"<br>"social forest*"<br>"local forest*"<br>"commons forest*"<br>"town forest*"<br>"city forest*"<br>"metropolitan forest*"<br>"suburban forest*"<br>"nonurban forest*"<br>"nonmetropolitan forest*"<br>"community woodland*"<br>"communal woodland*"<br>"community-managed woodland*"<br>"social woodland*"<br>"local woodland*"<br>"commons woodland*"<br>"town woodland*"<br>"urban woodland*"<br>"city woodland*"<br>"metropolitan woodland*"<br>"suburban woodland*"<br>"nonurban woodland*"<br>"nonmetropolitan woodland*"<br>"forest commons"	health<br>well-being<br>wellbeing<br>prosper*<br>"economic opportunit*"<br>"economic contribution*"<br>"economic impact*"<br>"economic benefit*"<br>livelihood*<br>"ecosystem service*"

TS=("community forest*" OR "communal forest*" OR "community-managed forest*" OR "social forest*" OR "local forest*" OR "commons forest*" OR "town forest*" OR "city forest*" OR "metropolitan forest*" OR "suburban forest*" OR "nonurban forest*" OR "nonmetropolitan forest*" OR "community woodland*" OR "communal woodland*" OR "community-managed woodland*" OR "social woodland*" OR "local woodland*" OR "commons woodland*" OR "town woodland*" OR "urban woodland*" OR "city woodland*" OR "metropolitan woodland*" OR "suburban woodland*" OR "nonurban woodland*" OR "nonmetropolitan woodland*" OR "forest commons")

AND

TS=(health OR well-being OR wellbeing OR prosper* OR "economic opportunit*" OR "economic contribution*" OR "economic impact*" OR "economic benefit*" OR livelihood* OR "ecosystem service*")

AND

((TS=("United States" OR USA OR U.S.A. OR U.S. OR Appalachia* OR "Great Lakes" OR mid-Atlantic-state* OR mid-Atlantic-region* OR middle-Atlantic-state* OR middle-Atlantic-region* OR Midwest*-US OR Midwest*-U.S. OR Midwest*-USA OR Midwest*-U.S.A. OR Midwest*-state* OR Midwest-state* OR "Great Plains" OR heartland OR "New England" OR Northeast*-US OR Northeast*-U.S. OR Northeast*-USA OR Northeast*-U.S.A. OR Northeast-state* OR northeast-state* OR "Pacific Northwest" OR Northwest*-US OR Northwest*-U.S. OR Northwest*-USA OR Northwest*-U.S.A. OR northwest*-state* OR Pacific-state* OR Southeast*-state* OR Southeast*-region OR Southeast*-US OR Southeast*-U.S. OR Southeast*-USA OR Southeast*-U.S.A. OR southern-state* OR southern-US OR southern-U.S. OR southern-USA OR southern-U.S.A. OR southwest*-state* OR southwest*-US OR southwest*-U.S. OR southwest*-USA OR

southwest*-U.S.A. OR "deep South" OR "Black Belt" OR "Rust Belt" OR "District of Columbia" OR "Washington DC" OR Washington-D.C. OR Alabama OR Alaska OR Arizona OR Arkansas OR "Little Rock" OR California OR "Los Angeles" OR "San Diego" OR "San Francisco" OR Colorado OR Connecticut OR Delaware OR Florida OR Gainesville OR Jacksonville OR Tampa OR Tallahassee OR Georgia OR Atlanta OR Hawai* OR Honolulu OR Idaho OR Illinois OR Chicago OR Indiana OR Indianapolis OR "West Lafayette" OR Iowa OR Kansas OR Wichita OR Kentucky OR Louisiana OR "New Orleans" OR "Baton Rouge" OR Shreveport OR Maine OR Orono OR Maryland OR "johns Hopkins" OR Massachusetts OR Boston OR Harvard OR Michigan OR Detroit OR "Ann Arbor" OR "East Lansing" OR Minnesota OR Minneapolis OR Rochester OR Mississippi OR Missouri OR Montana OR Missoula OR Nebraska OR Nevada OR "Las Vegas" OR "New Hampshire" OR "New Jersey" OR "New Mexico" OR "New York" OR "North Carolina" OR "North Dakota" OR Ohio OR Cincinnati OR Oklahoma OR Oregon OR Pennsylvania OR Philadelphia OR "Rhode Island" OR "South Carolina" OR "South Dakota" OR Tennessee OR Nashville OR Memphis OR Texas OR Houston OR Utah OR Vermont OR Virginia OR Seattle OR "West Virginia" OR Wisconsin OR Wyoming))
OR (TI=(Washington)) OR (AB=(Washington)) OR
(PS=(Washington)) OR (AD=((Birmingham AND "AL") OR Huntsville OR (Montgomery AND AL) OR Anchorage OR Fairbanks OR Phoenix OR Tucson OR Flagstaff OR Berkeley OR Stanford OR Vail OR Denver OR Farmington OR "New Haven" OR Hartford OR Wilmington OR Newark OR Miami OR (Athens AND GA) OR (Augusta AND GA) OR Boise OR Urbana OR Evanston OR Lexington OR Louisville OR Bardstown OR (Scarborough AND ME) OR Bethesda OR Baltimore OR Rockville OR (Worcester AND MA) OR Burlington OR "St Paul" OR "Saint Paul" OR (Jackson AND MS) OR (Columbia AND MO) OR Bozeman OR Omaha OR Lincoln OR Columbus OR Cleveland OR Portland OR Hershey OR Providence OR Richmond OR Washington))**)**

Running note: Run string from the Advanced Search. Set date limits on Advanced Search page or in the results sidebar.
